# Gamma delta T-cell-based immune checkpoint therapy: attractive candidate for antitumor treatment

**DOI:** 10.1186/s12943-023-01722-0

**Published:** 2023-02-15

**Authors:** Zhifei Gao, Yifeng Bai, Anqi Lin, Aimin Jiang, Chaozheng Zhou, Quan Cheng, Zaoqu Liu, Xin Chen, Jian Zhang, Peng Luo

**Affiliations:** 1 The Department of Oncology, Zhujiang Hospital, Southern Medical University, 253 Industrial Avenue, Guangzhou, Guangdong 510282 People’s Republic of China; 2 The Second Clinical Medical School, Zhujiang Hospital, Southern Medical University, Guangzhou, 510282 People’s Republic of China; 3 The Department of Oncology, Sichuan Provincial People’s Hospital, University of Electronic Science and Technology of China, Chengdu, 611731 China; 4 The Department of Urology, Changhai hospital, Naval Medical University (Second Military Medical University), Shanghai, China; 5 The First Clinical Medical School, Southern Medical University, Guangzhou, China; 6 The Department of Neurosurgery, Xiangya Hospital, Central South University, Changsha, Hunan China; 7 National Clinical Research Center for Geriatric Disorders, Xiangya Hospital, Central South University, Changsha, China; 8 The Department of Interventional Radiology, The First Affiliated Hospital of Zhengzhou University, Zhengzhou, Henan China; 9 The Department of Pulmonary and Critical Care Medicine, Zhujiang Hospital, Southern Medical University, Guangzhou, Guangdong China

**Keywords:** γδT cells, Immune checkpoint molecules, Immune checkpoint inhibitors (ICIs), Immune checkpoint blockade (ICB), Checkpoint inhibitor (CPI), Tumor microenvironment (TME), Immune checkpoint therapy (ICT), Antitumor immunotherapy

## Abstract

As a nontraditional T-cell subgroup, γδT cells have gained popularity in the field of immunotherapy in recent years. They have extraordinary antitumor potential and prospects for clinical application. Immune checkpoint inhibitors (ICIs), which are efficacious in tumor patients, have become pioneer drugs in the field of tumor immunotherapy since they were incorporated into clinical practice. In addition, γδT cells that have infiltrated into tumor tissues are found to be in a state of exhaustion or anergy, and there is upregulation of many immune checkpoints (ICs) on their surface, suggesting that γδT cells have a similar ability to respond to ICIs as traditional effector T cells. Studies have shown that targeting ICs can reverse the dysfunctional state of γδT cells in the tumor microenvironment (TME) and exert antitumor effects by improving γδT-cell proliferation and activation and enhancing cytotoxicity. Clarification of the functional state of γδT cells in the TME and the mechanisms underlying their interaction with ICs will solidify ICIs combined with γδT cells as a good treatment option.

**Supplementary Information:**

The online version contains supplementary material available at 10.1186/s12943-023-01722-0.

## Introduction

In recent years, many studies have shown that cytotoxic γδT cells have a strong killing ability toward autologous, allogeneic or xenogeneic tumor cells, and their concentration is often closely related to the clinical prognosis of patients [1–5]. In contrast to αβT cells, γδT cells can recognize antigens in a way that is not restricted by major histocompatibility complex (MHC) molecules [6], and they mediate the killing of target cells in various ways, participate in immunomodulation, and have a broader tumor cell killing spectrum [7, 8]. As the “bridge” between innate immunity and adaptive immunity as well as the first line of defense against tumors, γδT cells play an important role in the occurrence and development of tumors and can be used to evaluate the prognosis of patients [8–10]. Therefore, immunotherapy based on γδT cells has been studied in many kinds of tumors [11, 12].

Although previous types of adoptive cell therapy and other treatment methods have been effective [11, 13], these types of treatments often have low efficiency, and the response rate is not high. The reason for this lack of efficacy may be that the tumor microenvironment (TME) inhibits effector γδT cells through various mechanisms, thus making γδT cells dysfunctional and aiding in tumor cell immune escape [4, 14, 15]. The safety of Vγ9Vδ2T cell immunotherapy in practical applications has been partially confirmed, but only 10% ~ 33% of patients with hematological and solid malignant tumors benefit from this therapy [16]. In some cancer patients, the number and function of γδT cells is decreased, and the infiltrating γδT cells show an increased apoptosis rate, limited antitumor ability and an exhausted immunophenotype; these shortcomings can be improved by immune checkpoint inhibitor (ICI) treatment [17–19]. These findings indicate that some TMEs are immunosuppressive, which affects the normal function of γδT cells. The inhibition of γδT cells in the TME is the main factor that hinders their excellent antitumor ability. Tumor cells can undergo tumor immunoediting [20–23] and downregulate the expression of MHC-I molecules [24]; at the same time, immune checkpoint ligands (ICLs) are highly expressed. Binding with their respective immune checkpoint receptors (ICRs) leads to phosphorylation of the immune receptor tyrosine-based inhibition motif (ITIM)/immune receptor tyrosine-based switch motif (ITSM), leading to the activation of Scr homology region 2 domain-containing phosphatase (SHP-1/2), triggering a series of dephosphorylation events, and inhibiting cell activation [25]. To facilitate the immune escape of tumor cells, ICRs can inhibit cell activation by competing for ligands that bind to activated receptors [26, 27]. Therefore, it is very important to overcome the dysfunction of γδT cells in the inhibitory TME.

Targeting immune checkpoints (ICs) and reversing the inhibitory effects of the TME on cytotoxic γδT cells is the key to the success of immune checkpoint therapy (ICT). ICT has become the core pillar of tumor immunotherapy [28–30]. Studies have shown that the antitumor function of γδT cells can be recovered after ICIs are used [31]. In this paper, we hope that by describing the relationship between γδT cells, the TME and ICs, we can further reveal the antitumor potential of γδT cells and lay a theoretical foundation for the application of γδT cells in ICT.

## Interaction between the TME and γδT cells

**Fig. 1:**
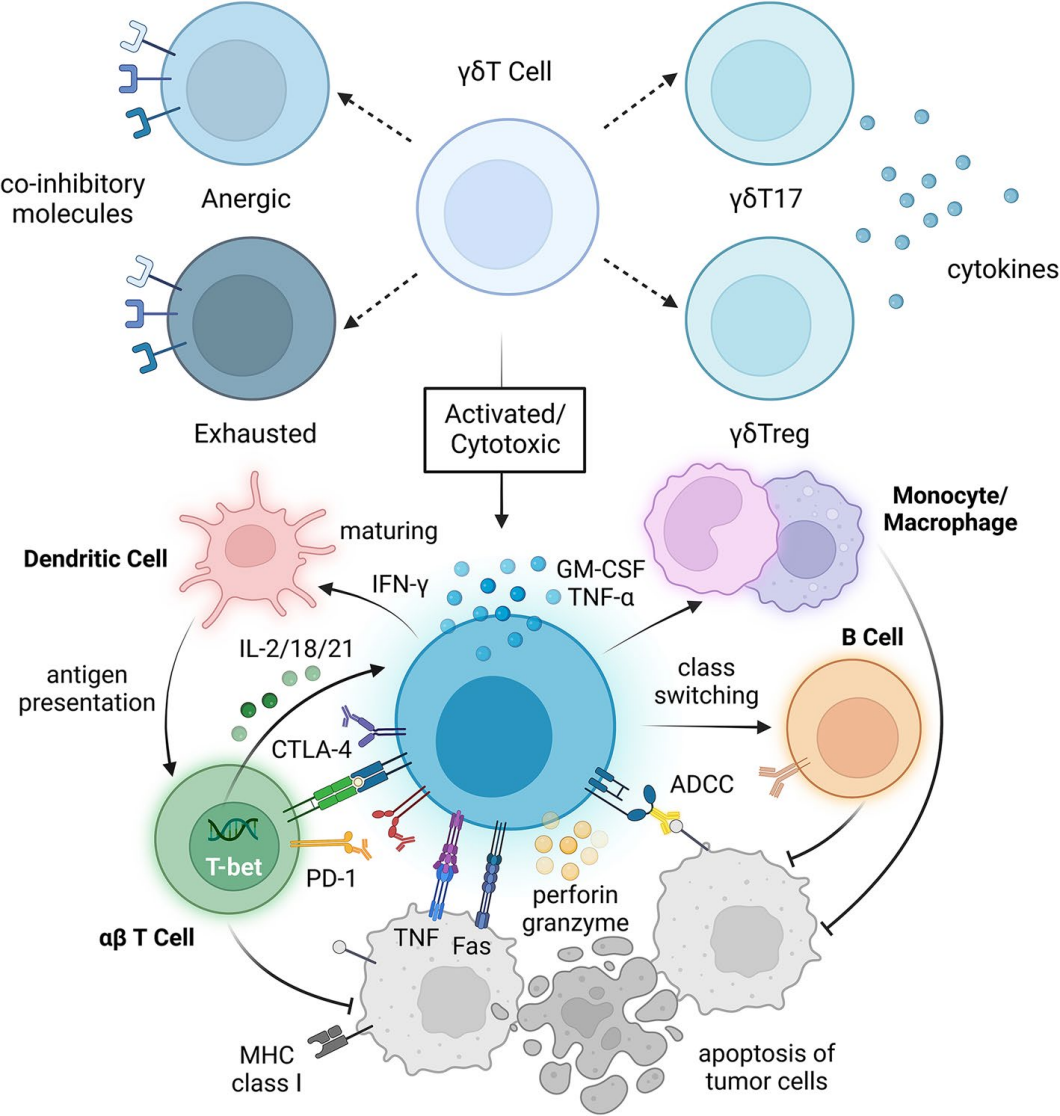
Under ideal conditions, activated γδT cells can kill tumor cells. Depending on the cytokines secreted by γδT cells and the microenvironment in which they are located, γδT cells can differentiate into different subpopulations, such as γδT17 or γδTregs. To some extent, the expression of checkpoint molecules on the surface of γδT cells can reflect their functional status. Activated effector γδT cells are cytotoxic, and they can express Fas ligand (FasL) and tumor necrosis factor-related apoptosis-induced ligand (TRAIL). Through direct cell–cell contact, apoptosis of tumor cells can be induced through the Fas-FasL and TRAILR-TRAIL death receptor pathways. Proinflammatory cytokines such as IFN-γ and TNF-α can directly inhibit tumor cells, and perforin-granzyme can directly act on the target cell membrane, leading to tumor cell cytolysis. αβT cells can promote the function of γδT cells through cytokines such as IL-2 and the transcription factors T-bet and have a synergistic antitumor effect with γδT cells. Dendritic cells and monocytes-macrophages can also be activated to promote antigen presentation and antibody class switching of B cells, as well as enhance the antibody-dependent cell-mediated cytotoxicity (ADCC) of γδT cells

γδT cells are a relatively rare population in peripheral blood (PB) T cells and are one of the main types of intraepithelial lymphocytes (IELs) of mucosal tissues [32]. γδT cells have many subgroups and high plasticity [33, 34] (Fig. S1–S4). According to differences in γ and δ chains, γδT cells can be divided into different structural subgroups. In the process of γδ T-cell receptor (TCR) rearrangement, Vδ2 is almost always coexpressed with Vγ9, so Vγ9VδT cells are the main type of γδT cells in human PB [35, 36]. Different functional subsets of γδT cells have diverse immune functions (Fig. 1). For example, depending on the cytokines they secrete and the microenvironment in which they are located, γδT17 cells, which mainly secrete interleukin (IL)-17, can have either immunosuppressive or immune-promoting properties; in an immunosuppressive microenvironment, the γδ regulatory T cell (γδTreg) subpopulation tends to play a role similar to that of αβTreg cells [37, 38]. In addition, the TME of tumor patients has a great influence on the phenotype and functional distribution of γδT cells [39, 40]. For example, the expression of ICs can be different in different γδT-cell subsets and may be determined by the stimulation of cytokines in the differentiation environment. PD-1 is evenly distributed across several subgroups with different functions in Vγ9Vδ2T cells, but compared with that in Vδ2 T cells, the proportion of TIGIT+, PD-1+ and TIM-3+ cells in Vδ1 T cells is higher [41, 42].

Many experimental and clinical studies have shown that γδT cells have antitumor effects [18, 43, 44]. High levels of γδT cells were positively correlated with clinical stage, overall survival (OS) time, and CD8+/CD4+ T-cell infiltration [45]. In metastatic colorectal cancer, γδT cells still show the ability to limit tumor progression [5]. By analyzing 25 kinds of cancers (other than brain cancers), it was found that a tumor-related γδT-cell gene signature was the factor most correlated with the OS rate of patients [46]. Furthermore, Vγ9Vδ2T cells can preferentially penetrate into basement membrane tissue, and preclinical studies have proven the ability of Vγ9Vδ2T cells to prevent brain tumor development [47, 48].

γδT cells are also involved in immunomodulation and indirectly exert antitumor effects mainly by interacting with other immune cells. For example, via interferon-gamma (IFN-γ), tumor necrosis factor-alpha (TNF-α), and granulocyte-macrophage colony stimulating factor (GM-CSF) production, γδT cells are able to induce the activation of macrophages or monocytes [49]. γδT cells can promote the differentiation and maturation of monocytes, and they can also interact with dendritic cells (DCs) via cell-to-cell contact or soluble molecules (especially I/II IFNs and BTN3A) [50], thus promoting cross-regulation and mutual activation between γδT cells and DCs. Plasmacytoid dendritic cells (pDCs) stimulated by Toll-like receptor 7 and TLR9 ligand (TLR7/9-L) or zoledronate (ZOL) can activate γδT cells, promote their proliferation, enhance their secretion of Th1 cytokines, and enhance their cytotoxic activity [50]. γδT cells can trigger phenotypic changes and functional activation of pDCs [50, 51]. γδT_APC_ cells can assist antigen presentation, promote expression of MHC-I molecules by tumor cells, and enhance the expression of costimulatory molecules such as CD80/86, thus activating DCs and αβT cells and playing an important role in the activation of B cells [52]. In addition, γδT cells can also express the chemokine receptor CXCR5, and after binding with CXCL13, γδT cells regulate the recruitment of B cells and antigen-presenting cells, secrete cytokines such as IL-4 and IL-10, and participate in immunoglobulin class switching and somatic hypermutation (SHM) [53, 54]. IL-4 plays an important role in the homotypic transformation of IgA and maintaining the structure of the germinal center (GC) [55]. Through various interactions with other immune cells, γδT cells can activate immunity together with effector cells and resist the growth of tumor cells (Fig. 1).

### Dysfunction of γδT cells in the TME

#### Continuous stimulation with tumor-associated phosphoantigens promotes the exhaustion of γδT cells

**Fig. 2:**
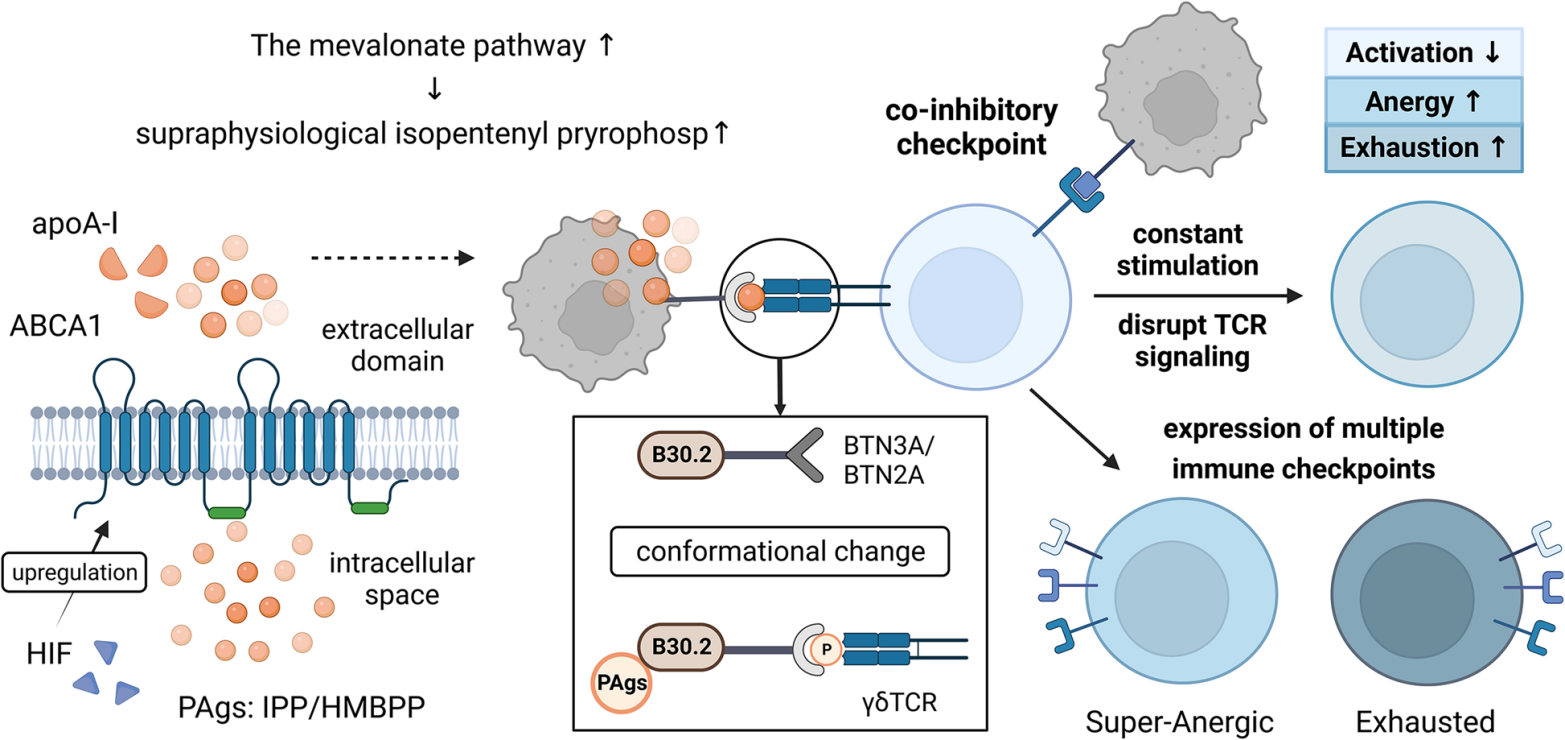
γδT cells gradually become dysfunctional in the tumor microenvironment. During tumorigenesis and tumor development, the metabolism of tumor cells is changed, and the phosphoantigens produced through the mevalonate pathway increase. ABCA1 and apoA-I can act synergistically with BTN proteins to stimulate γδT cells and induce the secretion of supraphysiological levels of IPP. Binding to the B30.2 domain causes the conformation of BTN3A/BTN2A to change, which promotes γδT-cell antigen recognition, proliferation and activation. At the same time, immune checkpoint ligands are highly expressed on the surface of tumor cells, which can interfere with the normal TCR signaling pathway and transmit inhibitory signals to γδT cells by binding to immune checkpoint receptors. Excessive and persistent antigen stimulation and the inhibitory signals transmitted because of the high expression of immune checkpoint molecules eventually cause γδT cells to enter an anergic or exhausted state, resulting in dysfunction and weakened antitumor effects

Due to the metabolic reprogramming of tumor cells, γδT cells gradually adapt to phosphoantigens (PAgs) in the TME, resulting in a low response state and ultimately exhaustion. PAgs usually originate from microbial I-4-hydroxy-3-methyl-but-2-enyl pyrophosphate (HMBPP) or endogenous isopentenyl pyrophosphate (IPP), and HMBPP can double the binding affinity between extracellular butyrophilin 3A (BTN3A) and Vγ9Vδ2 TCR [56]. During tumor cell development, the activity of the intracellular mevalonate pathway is enhanced, and the production of PAgs in the TME is upregulated and reaches the threshold for antigen stimulation of γδT cells under physiological conditions; thus, tumor cells can become the target of γδT cells [50, 57]. PAgs can rapidly induce Ca2+ signal transduction and activate Vγ9Vδ2T cells after cell-to-cell contact occurs [58]. The BTN gene is located in the MHC gene region of humans and is the key molecule involved in PAg-mediated activation of Vγ9Vδ2T cells [59–61]. BTNs have the following three gene subfamilies in humans: BTN1, BTN2, and BTN3 [62–64]. BTN3A2 is abundant in tumor tissues and has been proven to have a negative regulatory effect on natural killer (NK) cells [65]. Further study showed that there was an interaction between isomers, and BTN3A2 could regulate the subcellular transportation, localization and optimal activity of BTN3A1 [66]. Some scholars have hypothesized that BTN3A2 may affect receptor binding by binding to the hypothesized ligand, which may decrease antigen recognition sensitivity or lead to the exhaustion of γδT cells [65]. The activation of Vγ9Vδ2T cells is also related to members of the ABC transport family [50, 67, 68]. ABCA1 is thought to be the key molecule needed for PAgs to enter Vγ9Vδ2T cells before the B30.2 domain of BTN3A1 is activated [69]. The participation of transcription factors such as HIF-1α in the multiple myeloma TME promotes the active function of myeloid suppressor cells and Tregs and upregulates the expression of PD-L1 and ABCA1 on the surface of tumor cells. Then, ABCA1, apolipoprotein A-I (apoA-I) and BTN3A1 play a synergistic role causing myeloma cells to secrete supraphysiological levels of IPP, which eventually leads to the exhaustion of Vγ9Vδ2T cells and a decrease in cytotoxicity and other related antitumor effects [70] (Fig. 2).

#### Immune-related inhibitory molecules and cells reduce the antitumor effects of γδT cells

Immunosuppressive cells and related inhibitory molecules infiltrating the TME can promote the exhaustion of γδT cells and reduce the antitumor function of γδT cells. Myeloid-derived suppressor cells (MDSCs) in the TME can inhibit the secretion of IFN-γ by γδT cells, thus inhibiting their cytotoxic activity [71]. Mesenchymal cells, M2-type tumor-associated macrophages and Tregs can prevent the infiltration and/or activation of cytotoxic γδT cells through the synthesis and release of immunosuppressive molecules [72, 73]. Inhibitory neutrophils can affect the proliferation and activation of circulating γδT cells and the production of cytokines through reactive oxygen species (ROS). The increase in neutrophils and their related soluble mediators is related to a decrease in the survival rate of cancer patients [74–76].

**Fig. 3:**
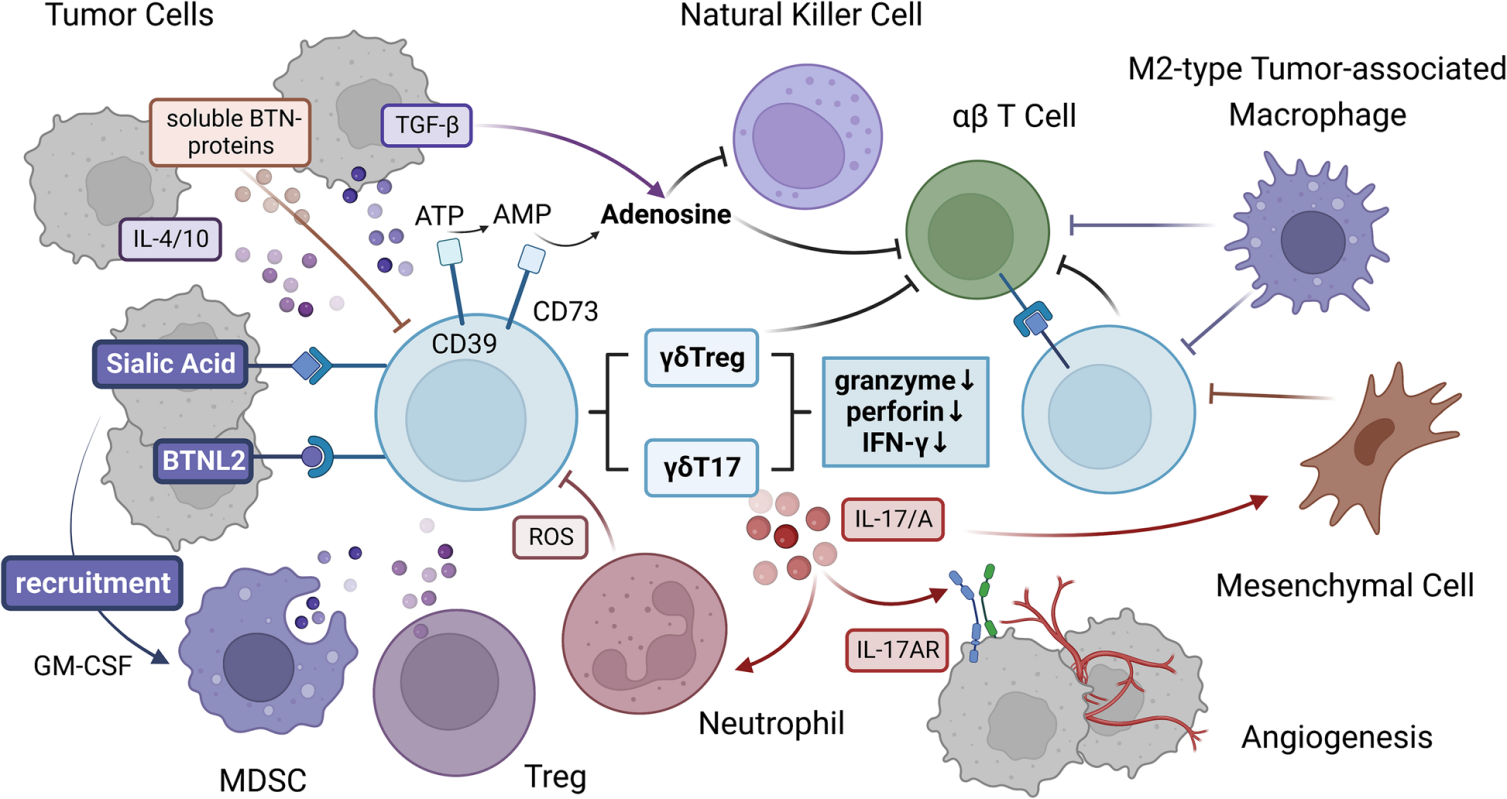
Crosstalk between γδT cells and the TME. Myeloid-derived suppressor cells (MDSCs), regulatory T cells (Tregs), neutrophils with a suppressive phenotype and related immunosuppressive molecules in the tumor microenvironment (TME) can inhibit γδT cells. The binding of glycoimmune checkpoint molecules and BTNL2 to their receptors can lead to the recruitment of MDSCs. Under the action of some cytokines, such as TGF-β, IL-4, and IL-10, in the TME, γδT cells can differentiate into cells with a suppressor phenotype and upregulate the expression of CD39/CD73, which can promote γδT cell exhaustion. γδT cells gradually develop dysfunctional characteristics, such as decreased secretion of perforin, granzyme and IFN-γ; in contrast, γδT cells secrete IL-17/A, which promotes tumor development and even metastasis by increasing angiogenesis and the infiltration of other inhibitory immune cells. At the same time, γδT cells express high levels of immune checkpoint ligands and inhibit other immune effector cells, such as αβT cells, by binding to immune checkpoint receptors on their surface. In addition, CD39/CD73, as the rate-limiting enzyme of the adenosine pathway, can promote the accumulation of adenosine in the TME by acting with soluble molecules such as TGF-β and the sBTN proteins. These interactions generate an immunosuppressive network by inhibiting effector cells, including but not limited to γδT cells, αβT cells and NK cells, through adenosine receptors, which further reduces the antitumor effects of γδT cells

Under the action of some cytokines, γδT cells can differentiate into other inhibitory phenotypes, and their antitumor function is reduced. IL-10 and transforming growth factor-β (TGF-β) in the TME can promote the transformation of γδT cells into tumor-promoting phenotypically distinct functional subgroups, such as γδT17 cells and γδTregs [72, 77]; in addition, inhibitory γδT cells tend to express CD39 and CD73 molecules [78–80], which can be signs of T-cell dysfunction [81–83]. For example, in breast tumors, the TME of tumor-bearing mice can induce γδT cells to differentiate into γδT17 cells and produce IL-17 [84]; when the production of IL-17 by Vδ1+ T cells in the lung increases, it promotes the infiltration of inhibitory neutrophils into lung adenocarcinoma and the development of tumors [85, 86]. CD39/CD73+ tumor-infiltrating lymphocytes (TILs) are defined as early dysfunctional T cells. They have significantly decreased secretion of IFN-γ and IL-2 and expression of perforin and granzyme, in addition to significantly increased expression of PD-1. Ultimately, these cells can hinder the activation of γδT cells mediated by PAgs and TCR [87]. Tumor-derived TGF-β is able to induce CD39/CD73+ γδT cells to differentiate into immunosuppressive T cells, which promote γδT cell dysfunction (Fig. 3).

#### Abnormal IC signaling promotes γδT-cell dysfunction

High expression of ICLs was observed in tumor cells, and aberrant signaling tends to cause anergy or exhaustion of γδT cells. The level of ICRs on the surface of γδT cells is higher than that of normal cells, which results in dysfunction and decreased cytotoxicity. Competition between inhibitory and costimulatory signals leads to γδT-cell anergy. Some ICs, such as PD-1, CTLA-4, and BTLA, can block TCR signaling and interfere with costimulatory signaling, ultimately blocking the activation of γδT cells and leading to γδT-cell dysfunction. Butyrophilin-like protein 2 (BTNL2) is highly expressed in many human tumor samples, BTN/BTNL can bind to the Vγ chain in γδTCR, and the interaction may affect TCR signal transduction [88, 89]. Castella et al. [90] found that the upregulation of the expression of multiple checkpoints, such as PD-1, TIM-3 and LAG-3, on γδT cells can even lead to a state of “super anergy”.

Prolonged peak expression and increased numbers of different ICs lead to the exhaustion of γδT cells. PD-1 expression, which is associated with methylation of the promoter DNA sequence, peaks 2 to 4 days after TCR activation in adult Vδ2 cells, but the peak expression remains longer in neonatal Vδ2 cells [91], after which it gradually drops to a moderate level [92]. However, γδT cells in the immune microenvironment mostly continuously express high levels of PD-1, suggesting that decreased cytotoxicity of γδT cells is related to overexpression of PD-1. PD-1 expression in Vδ2T cells is significantly increased in hepatocellular carcinoma [93], and LAG-3 is highly expressed in dysfunctional γδT cells [4, 94]. Double negative (DN) T cells infiltrating Merkel cell carcinoma are mainly composed of Vδ2T cells expressing PD-1 and LAG-3, which is consistent with the phenotype of exhausted and immunosuppressed γδT cells [95]. The expression of PD-1, TIM-3 and TIGIT may lead to the exhaustion or dysfunction of γδT cells in patients with acute myelogenous leukemia (AML) and multiple myeloma (MM) [31]. The proportion of TIGIT+CD226– γδT cells is increased in AML patients [96], which suggests that increased expression of TIGIT and significantly decreased CD226 expression may be associated with γδT-cell dysfunction. The frequency of TIM-3 + Vδ2 T cells in AML patients was significantly higher than that in healthy controls, and the levels of PD-L1 and high mobility group box protein 1 (HMGB-1) were also higher [97]. Vδ2T cells highly express BTLA in the lymph nodes of lymphoma patients [98], and the circulating soluble BTLA (sBTLA) level can be used as an independent prognostic factor for the OS of patients after ICI treatment [99]. The expression of PD-1 and TIM-3 on CD39 + γδT cells in laryngeal carcinoma was significantly higher than that in paired normal tissues [100]. Furthermore, the expression of B7-H3 in PB and tumor tissues of colon cancer patients was also significantly increased, and the proportion of circulating tumor-infiltrating γδT cells expressing LAG-3 in melanoma patients was increased, indicating that LAG-3 may help tumor cells achieve immune escape by inhibiting γδT cells [101].

In addition to traditional ICs such as PD-1, human leukocyte antigen (HLA) class I inhibitory receptors also transmit inhibitory signals that interfere with the effector function of γδT cells. The downregulation of β2M gene expression in tumor cells leads to the downregulation of MHC-I molecule expression on the cell surface and high expression of HLA-G, which can decrease the clonality of Vγ9Vδ2T cells and the secretion of IFN-γ because HLA-G can bind to inhibitory leukocyte immunoglobulin-like receptors (LIRs) or Ig-like transcripts (ILTs), such as ILT2 [102–105]. After blocking the inhibitory signal transmitted by ILT2, γδT-cell proliferation and cytotoxicity are improved [104, 106]. Similarly, inflammatory factors in the TME tend to lead to overexpression of HLA-E, and the expression level is even higher than that of PD-L1. Furthermore, the continuous stimulation of TME-derived cytokines such as TGF-β and IL-15 and tumor antigens also upregulates the expression of CD94/NKG2A [61, 107]. In NK cells, NKG2A can inhibit cytotoxic effector function by disrupting the actin network at the immune synapse of the activating receptor NKG2D [108]. In γδT cells, NKG2A receptor-mediated inhibitory signaling tends to prevail [109]. NKG2A+ and NKG2A- cells can be formed from γδT cells during early thymic development and do not appear to change significantly with cell growth [110], and their expression kinetics are similar to those of cells expressing TIM-3 and CD39, which emerge late but are stably expressed [111]. The combination of HLA-E with NKG2A, dependent on the participation of ITIMs and SHP1/2, can transmit inhibitory signals to interfere with the activation of γδT cells and limit the function of effector γδT cells. NKG2A + Vδ2 T cells in glioblastoma multiforme (GBM) have been shown to affect the OS of patients. Unlike its role in healthy tissues, HLA-E in tumor tissues significantly regulates the high reactivity of NKG2A + Vδ2 TILs, ultimately generating dysfunctional immune cells and tumor cell escape [110].

Glycoimmune checkpoints can also interfere with activation signals and transmit inhibitory signaling to γδT cells. Abnormal levels of carbohydrates are an important feature of cancer cells [112–114]. Many glycosyltransferases, including sialyltransferase, are often altered in malignant tumor cells, resulting in abnormal expression of sialoglycan [115]; high expression of sialoglycan can be recognized by sialic acid binding immunoglobulin-like lectin (Siglecs). Siglecs, which are highly expressed in TILs, can also transmit negative signals through ITIMs and SHP1/2, thus hindering the effector function of γδT cells. Tumor marker gangliosides (TMGs) can interfere with the binding of IL-2 and IL-2R, and sialylation of cancer cells can destroy the interaction between NKG2D and ligands, hinder the transmission of activation signals, and inhibit the proliferation and activation of T cells [116]. Galectins, members of the lectin family, are also regulatory glucose checkpoints. Gal-1 can interact with TCR and CD45, affect CD45 phosphatase activity and the downstream signaling pathway of effector T cells, and selectively promote Treg amplification and secretion of IL-10, eventually leading to T-cell exhaustion and promoting tumor cell immune escape [117]. Gal-9 is a ligand of TIM-3, which can affect the TCR activation threshold by binding to N-glycans and selectively binding to PD-1 in a manner mediated by glycans [118]. Blockade of Gal-3, a ligand of LAG-3, can reverse the exhaustion of T cells [119]. Furthermore, the P-selectin ligand PSGL-1 can inhibit antitumor immunity by interacting with the IC VISTA [119]. In addition, glycoimmune checkpoint molecules can act on myeloid regulatory cells. For example, MUC1 can bind to Siglec-9 on macrophages, leading to their differentiation into immunosuppressive M2 macrophages and upregulating the expression of PD-L1 [120]. It has been proven that decreasing the level of sialoglycan in melanoma and neuroblastoma mice with Ac53FaxNeu5Ac can inhibit tumor growth and significantly change the immune cell composition of the TME [121].

### Effects of γδT cells on the TME

Studies have found that in addition to high expression of ICRs, exhausted γδT cells often show high expression of ICLs. When ICLs bind with ICRs on other immune effector cells, they provide inhibitory signals and negatively regulate the killing function of T cells [86, 122]. Preclinical data suggest that infiltrating γδT cells account for 40% of the infiltrating lymphocytes in pancreatic ductal adenocarcinoma (PDAC), and high expression of PD-L1 in these γδT cells inhibits the activation of other T cells [123]. PDAC tumor growth was inhibited in the presence of anti-PD-L1 antibodies, but anti-PD-L1 antibodies had no effect on tumor growth in mice lacking γδT cells (TCRγδ−/−) [124], highlighting the importance of the interaction between γδT cells and the TME.

Once activated in the TME, γδT17 cells can secrete immunosuppressive cytokines, promote the expression of PD-L1 and participate in the construction of an inhibitory TME [114, 125]. Driven by BTNL2, γδT17 cells secrete IL-17A and recruit myeloid suppressor cells, resulting in an increase in the number of TME mesenchymal stem cells, M2 macrophages and tumor-associated macrophages (TAMs) [126]. It has been confirmed that the main cell group producing IL-17A in laryngeal carcinoma is CD39 + Vδ1 T cells [100]. IL-17 supports tumor growth during early tumorigenesis [127], in addition to recruiting immunosuppressive cells, promoting angiogenesis and even directly promoting the proliferation and metastasis of tumor cells [128–130]. IL-17 also leads to granulocyte colony stimulating factor (G-CSF)-mediated tumor-associated neutrophil expansion; blocking CCL2 in mice reduces the production of IL-17 by γδT17 cells, thus reducing the proliferation of neutrophils and enhancing the activity of CD8+ T cells. IL-17 also promotes the secretion of IL-6, activating the STAT3 pathway and inducing the expression of PD-L1 [131]. Furthermore, Vδ1T17 cells also secrete IL-8, IL-17 and GM-CSF to recruit immunosuppressive cells such as MDSCs to establish an immunosuppressive network.

High expression of CD39 and CD73 on the surface of dysfunctional γδT cells can inhibit other effector cells through the adenosine pathway. CD39 can hydrolyze eATP into adenosine monophosphate (AMP), while CD73 can convert AMP into immunosuppressive adenosine (ADO) [132, 133]. ADO inhibits antitumor immunity through the A2A receptor (A2AR) expressed on immune cells. A2AR can block the early signal transduction of TCR in a cyclic AMP (cAMP)-dependent manner, thus weakening the response of T cells and inhibiting the proliferation and the cytokine production of T cells; the A2AR antagonist CPI-444 can reverse the suppression of T-cell signal transduction and IL-2 and IFN-γ secretion [134]. Approximately 20% of the infiltrating γδT cells in human advanced breast cancer samples express CD73, and these cells may play an immunosuppressive role by producing immunosuppressive molecules such as IL-10, IL-8 and ADO, thus promoting tumor growth [135]. Regulatory CD39+ γδT cells inhibit the proliferation of other effector T cells in a concentration-dependent manner [80]. CD39 and CD73 are closely related to the prognosis of patients [136]. Moreover, researchers have confirmed that CD39+ γδTregs can directly inhibit effector T cells more strongly than CD4+ or CD8+ Tregs through the ADO pathway [137]. The inhibitory function of CD39 and CD73 is not particularly affected by the use of monoclonal antibodies (mAbs) that block CTLA-4 or PD-1 [137, 138].

## Targeting ICs can regulate the immune function of γδT cells

In the inhibitory TME, γδT cell dysfunction develops gradually. It is difficult to reverse for terminal exhaustion of γδT cells by antigen re-exposure or inhibition signal blockade, and dysfunctional γδT cells may even aid tumor cells [139–143]. Therefore, early intervention and reversal of the state of immunosuppressive γδT cell state are key to the success of ICT. Targeting ICs on the surface of γδT cells can reverse the immunosuppressive state of γδT cells or revive exhausted γδT cells.

### Targeting BTN family proteins can affect the immune response of γδT cells

BTN and BTNL family proteins play an important role in antitumor immunity dominated by γδT cells and are also related to clinical prognosis. In non-small cell lung cancer (NSCLC) and breast cancer, the expression of BTN1A and members of the BTNL family is significantly related to OS [144]. Overexpression of BTN2/3A is associated with poor prognosis in glioma [145]. In addition, in many tumor models, high expression of BTN3A in tissues and high levels of soluble BTN3A2 and BTN2A in plasma can be used as markers of the efficacy of Vγ9Vδ2 T-cell immunotherapy [65, 146–151]. BTN3A2 can also regulate the interaction between γδT-cell receptors and the NF-κB signaling pathway [148], which is related to the survival time of patients [152]. Studies have shown that specific BTNs have spatiotemporal specificity in shaping or selecting γδT-cell subsets [153]. Therefore, it is very important to further characterize the interaction between γδT cells and BTNs.

BTN3A/CD277 is an indispensable molecule in γδT-cell activation. Administration of a mAbs that activated BTN3A1 can enhance the activation of γδT cells and their cytotoxic effects [63]. By adding anti-CD277 mAbs and knocking down CD277, Harly et al. showed that CD277 plays a unique role in the response of Vγ9Vδ2T cells induced by PAgs, and the mechanism of γδT-cell activation depended on CD277 and was based on TCR signal transduction [154]. Without any other stimulation (such as anti-CD3 mAbs), administration of the soluble mAb 20.1 targeting BTN3A/CD277 indirectly increased the antigen recognition of Vγ9Vδ2T cells and mimicked PAgs to induce complete activation of Vγ9Vδ2T cells, accompanied by activation of Ca2+ signal transduction, upregulation of CD69 expression and increased IFN-γ secretion [154, 155]. The anti-BTN3A mAb 20.1 combined with Vγ9Vδ2T-cell immunotherapy restored the proliferation and cytotoxicity of Vγ9Vδ2T cells in tumors, prevented the exhaustion of adoptively transferred Vγ9Vδ2T cells, improved the survival rate of animals and reduced the tumor burden in blood and bone marrow [152]. Aude De Gassart and coworkers [156] also developed a new humanized anti-BTN3A mAb, ICT01, that can specifically activate Vγ9Vδ2T cells and upregulate the proportion of CD69+ Vγ9Vδ2T cells in a concentration-dependent manner. ICT01 can induce the degranulation of Vγ9Vδ2T cells and promote the production of IFN-γ, TNF-α, IL-8, IL-1β, monocyte chemoattractant protein 1 (MCP-1) and other proinflammatory cytokines. In addition, after the application of ICT01, the tumor growth rate of mice was obviously slowed. Importantly, the mAbs had no obvious effect on normal cells, highlighting the potential clinical value of the anti-BTN3A mAb ICT01.

After blocking BTN proteins, the response of γδT cells to PAgs was blocked, and there was no longer a tumor cell killing effect. Yamashiro et al. found that the expression level of BTN3 was inversely correlated to the activity of lymphocytes, and administration of the mAb 232-5 lead to phosphorylation of the BTN3A3 molecule and transduction of negative signals; these effects caused CD4+ and CD8+ T cells to act like CD4 + CD25+ Tregs, accompanied by a decrease in cell proliferation and cytokine secretion [157]. However, the mAb 20.1 has no significant stimulatory or costimulatory effect on αβT cells [154], suggesting that BTN proteins may jointly inhibit effector T cells through negative signal transmission and that BTN3A3 may be a novel target [158]. In addition, although the mAb 103.2 does not affect the proliferation and activation of CD8+ T cells, it can inhibit the response of γδT cells induced by PAgs and even inhibit the degranulation and cytokine secretion of Vγ9Vδ2T cells, decreasing their antitumor effects. Audrey Benyamine and coworkers also demonstrated that the mAbs 103.2 and 108.5 targeting the BTN3A molecule can completely inhibit the tumor cell lysis mediated by Vγ9Vδ2T cells [65, 152]. In addition to BTN3A, the B30.2 domain of BTN2A is required for the response of γδT cells to PAgs. The expression of the BTN2A1/BTN3A1 complex can trigger the activation of Vγ9Vδ2TCR, and the expression of BTN2A1 in cancer cells is related to the cytotoxicity of Vγ9Vδ2T cells. Anti-BTN2A1 mAbs can significantly inhibit the degranulation of Vγ9Vδ2T cells, and the inhibition of Vγ9Vδ2T-cell cytotoxicity induced by the 7.48 mAb generates an effect similar to the real TME [159].

**Fig. 4:**
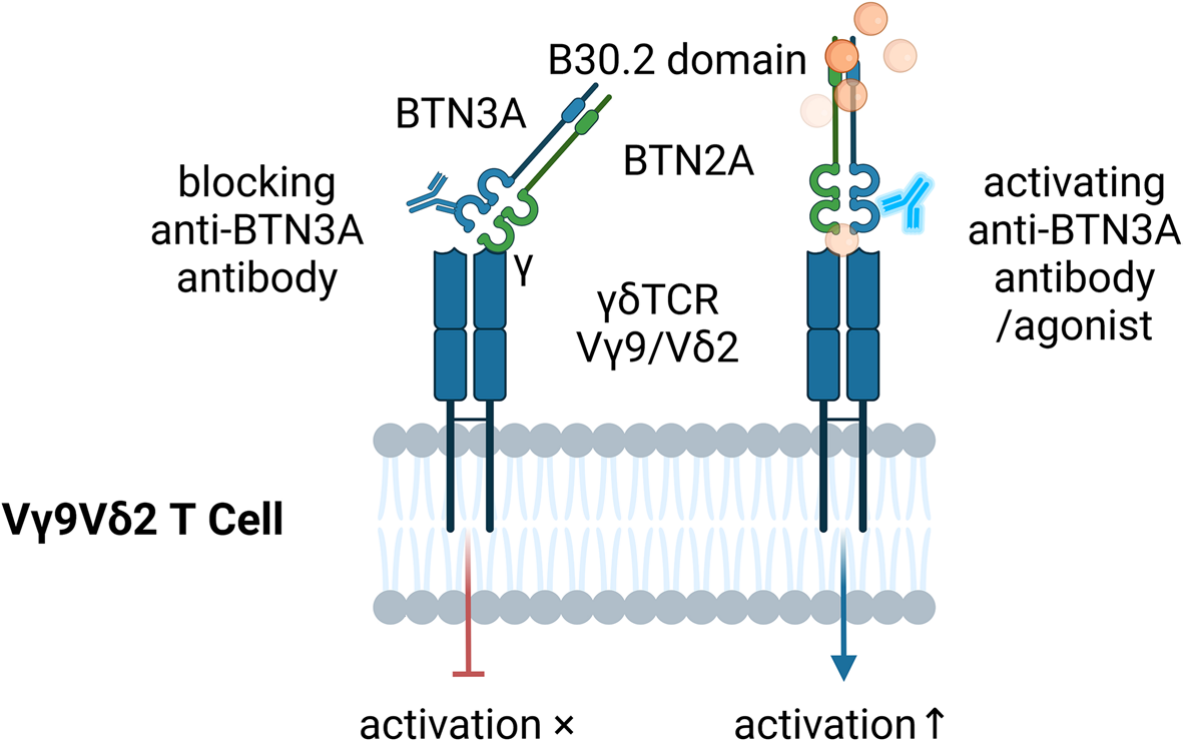
The activation of γδT cells can be modulated by anti-BTN3A antibodies. BTN2A can bind to the γ chain of γδTCR and plays an important role with BTN3A in the activation of γδT cells by phosphoantigens. After the binding of an antagonistic monoclonal antibody to BTN3A, the activation of Vγ9Vδ2T cells will be blocked, and the cytotoxicity of Vγ9Vδ2T cells will decrease or even disappear. In contrast, after the use of an agonist targeting BTN3A, Vγ9Vδ2T cells have increased antigen sensitivity and enhanced ability to kill tumor cells, suggesting that targeting BTN family proteins can significantly regulate the immune response of γδT cells against tumor cells

Targeting BTN family proteins is beneficial for restoring the antitumor function of γδT cells and promoting their interaction with other immune cells, and they exert their antitumor effects synergistically. It has been reported that melanoma cells can hijack the interaction between pDCs and γδT cells to escape immune control, which is manifested as dysfunction of BTN3A and impaired ability of γδT cells to regulate ICs [160]. Conformational change of BTN3A1 is the key event of PAg perception [161], and BTN2A1 is the key ligand that binds to the Vγ9+ TCR γ chain; it can directly bind to the germline coding region of the Vγ9 chain in Vγ9Vδ2TCR and simultaneously bind to BTN3A1 on the cell surface. The synergistic effect of BTN3A1 and BTN2A1 enhances the recognition of target cells by Vγ9Vδ2T cells and plays an important role in PAg perception [162–166]. BTN3A1 can also recognize the N-mannosylated oligosaccharide in the near-membrane domain of CD45 and anchor the CD45 dimer near TCR, which may physically block the interaction of TCR-peptide-MHC-I complexes (pMHC), thus effectively inhibiting the separation of CD45 molecules from immune synapses and ultimately inhibiting the functions of TCRs and αβT cells [167]. Therefore, CD277-specific antibodies can restore the effector activity of αβT cells and induce BTN3A activity to mediate the synergistic killing of BTN3A1+ tumor cells by αβT cells and γδT cells in a BTN2A1-dependent manner. In summary, BTN family proteins are potential targets for fully exploiting the potential of γδT cells in IC therapy (Fig. 4).

### Blocking inhibitory IC signaling can reverse the immunosuppressive state of γδT cells

#### ICIs can restore the proliferation and activation of γδT cells

**Fig. 5:**
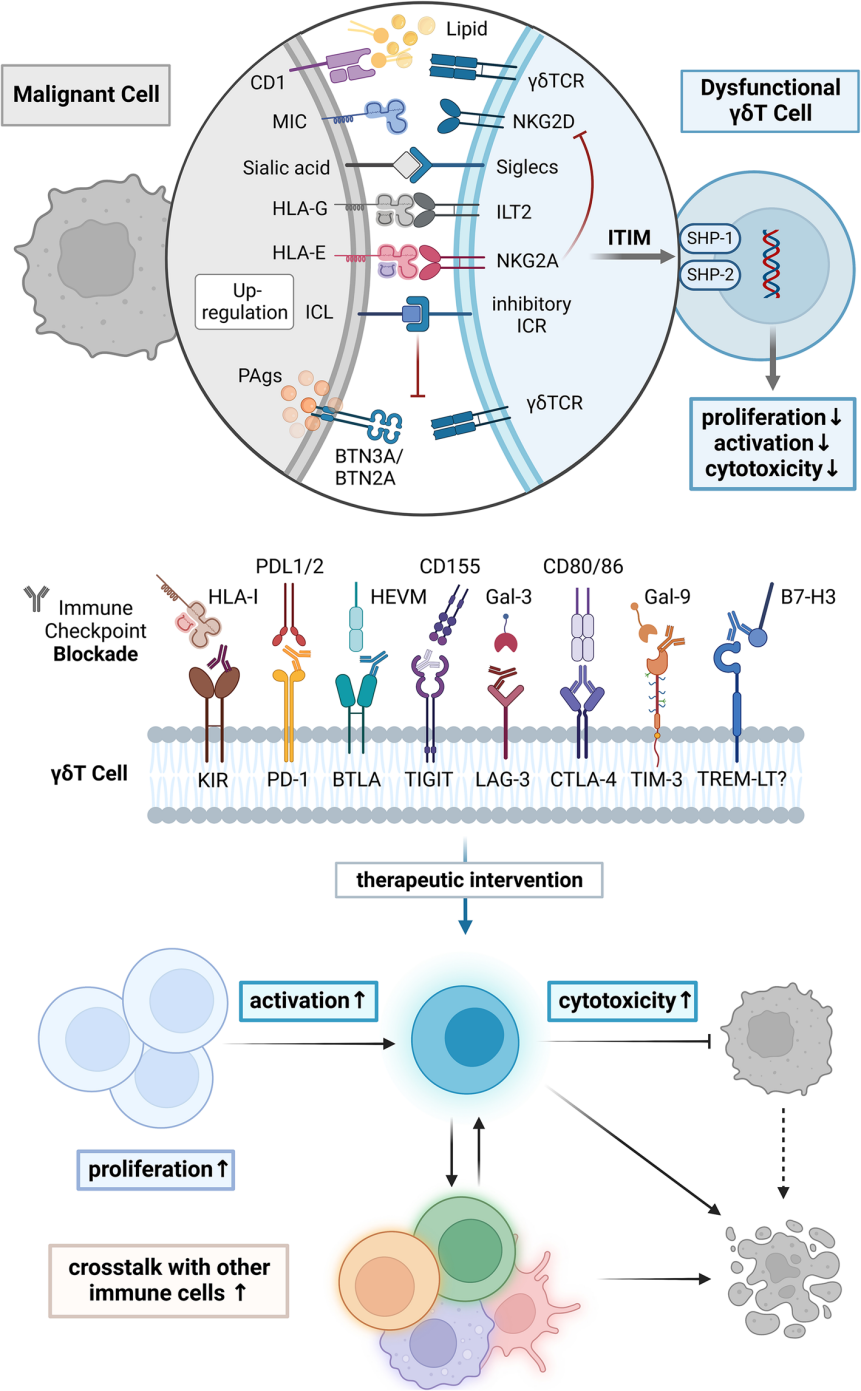
Targeting immune checkpoint molecules can revive dysfunctional γδT cells. Dysfunctional γδT cells express high levels of multiple checkpoint molecules on their surface, a phenotype similar to that of anergic or exhausted T cells. Vδ2T cells can recognize phosphoantigens with the assistance of BTN3A/BTN2A, and some subsets of Vδ1T and Vδ3T cells can recognize lipid antigens presented by CD1d and transmit activation signals through γδTCR. Activated γδT cells express NKG2D and/or other similar costimulatory molecules on their surface. Immunoglobulin-like transcripts (ILTs) or leukocyte immunoglobulin-like receptors (LIRs) belong to the Ig superfamily. ILT2 (LIRB1) binds to HLA-G in addition to recognizing other ligands and can inhibit the immune functions of γδT, NK, and B cells. NKG2A can recognize the nonclassical MHC-I molecule HLA-E and inhibit the stimulatory signal of NKG2D. Inhibitory Siglecs are immune regulatory sialic acid-binding receptors that resemble traditional immune checkpoint molecules with one or more ITIM-like motifs in the intracellular segment. Abnormal signaling of immune checkpoint molecules interferes with the normal function of TCRs, affects the level of intracellular protein phosphorylation through ITIM motifs and SHP-1/2, inhibits the proliferation and activation of γδT cells, and ultimately reduces the cytotoxicity of γδT cells. After blocking the inhibitory signals with monoclonal antibodies targeting immune checkpoint molecules, the ability of γδT cells to kill tumor cells and their interactions with other immune effector cells can be enhanced

The proliferation and activation of γδT cells were improved after administration of mAbs against ICs (Fig. 5). ICIs targeting B7-H3 and TIM-3 can improve the proliferation and/or activation of dysfunctional γδT cells. In colorectal cancer, knocking down or blocking B7-H3 can inhibit the apoptosis of Vδ2T cells, promote the proliferation of Vδ2T cells and induce the expression of the activation markers CD25 and CD69 in Vδ2T cells [168]. Caspase-3 participates in the activation of the TIM-3 signaling pathway, so γδT cells with upregulated TIM-3 are more prone to experience early apoptosis. After blocking TIM-3, the proliferation of Vγ9Vδ2T cells, the STAT phosphorylation level and the induction of IL-21 increased significantly [97, 169]. However, the expression of cyclin B1 and cyclin D1, which are related to cell proliferation, was not affected by TIM-3 blockade [170].

The expression of PD-1 may be one of the reasons for the failure of Vγ9Vδ2T-cell expansion in tumor patients. Blocking the PD-1 signaling pathway can partially restore the damaged proliferation of PD-1+ γδT cells and induce their activation. A study showed that the response of bone marrow-derived Vγ9Vδ2T cells to PAgs stimulation was weakened in MM patients, while the proliferation response of γδT cells was enhanced after zoledronic acid stimulation with anti-PD-1 mAbs in vitro [51]. In addition, researchers have found that the bispecific (PD-L1× CD3) antibody Y111, which can simultaneously recognize PD-L1 and CD3, can effectively connect T cells with tumor cells expressing PD-L1. In the presence of PD-L1+ tumor cells, Y111 can induce the activation of Vγ2Vδ2T cells in a dose-dependent manner [171]. Although some studies have shown that the inhibitory effects of the PD-1 signaling pathway on γδT cells may be reversed by the synergistic effects of TCR and IL-2 signaling, blocking PD-1 signal transduction with anti-PD-L1 antibodies alone does not affect IL-2 production by γδT cells [172, 173], and the inhibitory TME also weakens TCR signal transduction. Blocking the PD-1/PD-L1 signaling pathway, restoring the proliferation and activation of γδT cells and promoting their differentiation into antitumor effector cells in the early stage of γδT cell development are important for effective ICT.

Furthermore, some studies have shown that PD-1 is not the main molecule affecting the proliferation of γδT cells and that the proliferation of γδT cells is strictly regulated by BTLA [174]. BTLA and TCR are clustered at the synapse between Vγ9Vδ2T cells and target cells, and the close localization of BTLA and TCR suggests that BTLA may affect TCR-dependent signal transduction. γδT cells need TCR signals to maintain their stability [175], and it has been suggested that the interaction between BTLA and HVEM inhibits the proliferation of Vγ9VδT cells and their response to lymphoma cells. BTLA on PB γδT cells interacted with HVEM on leukemic cells and caused some cells to stagnate in S phase; however, this did not affect the percentage of G0 cells but did increase the percentage of cells in G2/M phase. After blocking the interaction between BTLA and HVEM with mAbs, PAg/TCR-mediated signal transduction can be enhanced, and the proliferation of γδT cells can be upregulated [51, 98, 176]. However, no synergistic effect was found after the combined blockade of BTLA and PD-1, suggesting that BTLA and PD-1 may have independent effects on the proliferation and cytotoxicity of human PB γδT cells [174]. That is, inhibition of the BTLA signaling pathway can promote the proliferation of γδT cells without affecting their cytotoxicity, the production of IFN-γ or the nontargeted degranulation induced by bromohydrin pyrophosphate (BrHPP) [176].

#### ICIs can enhance the cytotoxicity of exhausted γδT cells

After treatment with ICIs, the cytotoxicity of γδT cells was enhanced (Fig. 5). After blocking PD-1, the antibody-dependent cell-mediated cytotoxicity (ADCC) effects of CD16+ Vγ9T cells on lymphoma cells was improved [177]. However, it has also been reported that in the environment of PD-L1+ tumor cells, blocking or knocking out PD-1 does not significantly increase the cytotoxicity of γδT cells. In contrast, anti-PD-L1 mAbs could enhance the cytotoxicity of γδT cells against some cancer cells, and the expression level of PD-L1 was positively correlated with the cytotoxicity of γδT cells [178].

##### Increased secretion of antitumor cytokines

After ICs are blocked, γδT cells can produce more inflammatory cytokines, especially IFN-γ and TNF-α. Inhibitory ICs may reduce the production of IFN-γ by inhibiting the key transcription factor Eomes [179]. The PD-1 signaling pathway may be involved in the regulation of IFN-γ production by γδT cells [180]. An in vitro study of γδT cells showed that, similar to that of traditional αβT cells, the cytotoxicity of activated PD1+ Vδ2T cells was inhibited, and the secretion of IFN-γ decreased after PD-L1 binding [172]. Blocking PD-1 with antibodies such as pembrolizumab can promote the secretion and release of IFN-γ and TNF-α by activated γδT cells. After the combined use of anti-LAG-3 and anti-PD-1 antibodies, the secretion of cytokines, especially IFN-γ, in γδT cells increased [92, 171, 177]. However, it should be noted that presensitization of target cells or γδT-cell activation is needed for the production of IFN-γ; that is, the regulatory effects of PD-1 signaling on the proliferation and cytokine secretion of γδT cells depends on costimulatory signals or early activation of γδT cells [92]. The TIM-3 signaling pathway also inhibits the secretion of IFN-γ and TNF-α by γδT cells. In vitro studies have shown that TIM-3+ γδT cells do not produce IFN-γ or TNF-α and have reduced cytotoxicity [96, 181]. In addition, the effects of B7-H3 on the cytokine profile of Vδ2T cells was studied. It was found that B7-H3 inhibited the expression of IFN-γ in Vδ2T cells by inhibiting T-bet [168], suggesting that the ability of γδT cells to produce cytokines would be restored after blocking TIM-3 and B7-H3 with ICIs.

The expression of effector genes in the IFN signaling pathway is negatively correlated with the degree of γδT-cell exhaustion. In exhausted T cells, several inhibitory receptors, including VSIR, KLRG1, LAG3 and TIGIT, as well as the transcription factors NR4A2 and ID2, are significantly upregulated, and IFN response genes, such as IFITM1, STAT1 and IFI6, are also upregulated [182].

##### Increased secretion of perforin and granzyme

After blocking ICs, the expression of perforin and granzyme is not inhibited, and cytotoxicity is enhanced. In vitro studies showed that after a PD-1 blocking drug was used and ZA was administered as stimulation, the cytotoxicity of Vγ9Vδ2T cells increased nearly 5 times, accompanied by an increase in the expression of the degranulation marker CD107 and an increase in the proportion of CD107+ Vγ9Vδ2T cells [51]. ERK1/2, STAT-3 and Wnt are known to regulate the expression of perforin and granzyme B in various immune cells. Some studies have found that increased expression of members of the TIM-3 pathway can significantly decrease the level of pERK1/2 in Vγ9Vδ2T cells activated by recombinant human (rh) Gal-9 but does not affect the level of pSTAT3 or Wnt [183]. Therefore, blocking TIM-3 can increase the killing effects of Vγ9VδT cells on colon cancer cells by activating the ERK1/2 pathway and upregulating the expression of perforin and granzyme B. B7-H3 also inhibits the cytotoxicity of Vδ2T cells by downregulating the expression of perforin and granzyme B [168], which can be reversed by using B7-H3 blockers. In tumor tissue, compared with PD-1 + LAG-3– cells, PD-1 + LAG-3+ T cells have a weaker ability to produce cytokines and/or undergo degranulation [177]. The cytotoxicity of Vδ2T cells against NSCLC tumor cell lines was enhanced after blocking PD-1 [171, 174], so the degranulation of γδT cells can be improved with the inhibition of the PD-1/PD-L1 or LAG-3 signaling pathway.

KIRs increase the threshold for Vγ9Vδ2T cell antigen-based activation, inhibiting the killing effects of cytotoxic Vγ9Vδ2T cells on MHC-I+ tumor cell lines. Blocking the binding of NKG2A to HLA-E can restore the high responsiveness of NKG2A+ Vδ2T cells. Low expression of NKG2A is usually accompanied by high expression of other ICs, such as PD-1 [141]. NKG2A is often coexpressed with PD-1, CTLA-4, LAG-3 and TIM-3 in CD8+ T cells, but they have different inhibition mechanisms [184]. The safety of humanized anti-NKG2A mAbs has been verified in clinical trials, and studies on HLA-E+ tumor cells have proven that combinations blocking the inhibitory signals of PD-1 and NKG2A have synergistic effects, which are characterized by enhanced ADCC and increased expression of CD107 and degranulation substances [108, 185].

#### ICIs can promote synergistic antitumor effects of γδT cells and other immune cells

When ICLs on the surface of γδT cells are blocked, the positive interaction between γδT cells and other immune cells becomes more efficient (Fig. 5). Studies have shown that γδT cells are the key source of immunosuppressive ICLs in tumor tissues and may have the ability to regulate αβT cells. Blocking PD-L1 on γδT cells in PDAC can enhance the levels of infiltrating CD4+ and CD8+ T cells and improve immunotherapy efficacy [124]. Data from mouse models also indicate that specific γδT-cell subsets that express PD-L1 can inhibit αβT cell infiltration through PD-1/PD-L1 signaling and promote tumor growth. Vδ2T cells can also activate CTLA-4 and inhibit αβT cells through CD86. In individuals with normal γδT-cell function, the use of PD-L1 or Galectin-9 inhibitors can promote the expansion and activation of CD4+ and CD8+ T cells, but this effect is not observed in the absence of γδT cells [92, 124, 186]. In addition, PD-L1 is a downstream target of HIF1α [187]. Hypoxia and coculture with γδT cells increased the apoptosis rate of CD8+ T cells, suggesting that γδT cells can induce the death of CD8+ T cells, and this effect was significantly changed after blocking PD-1. After blocking BTNL2, the number of cytotoxic CD8+ T cells in the TME increased [126]. Monalizumab can inhibit the newly recognized IC NKG2A [188] and thus activate the antitumor effects of αβT, γδT and NK cells. Therefore, after using ICIs to reverse the immunosuppressive state of γδT cells, αβT cells can better promote tumor cell killing.

Targeting checkpoint molecules on γδT cells with ICIs can also inhibit Tregs. PD-L1 expressed by γδT cells can promote the production of Tregs and enhance the expression of the FOXP3 gene, thus maintaining the expression of TIGIT, which plays an important role in immune regulation; these effects promote Tregs to directly inhibit effector T cells through CTLA-4 and LAG-3 [71, 189]. A high TIGIT/DNAM-1 ratio was detected in Foxp3+ γδT cells of patients with AML and Tregs of patients with melanoma. High expression of TIGIT can promote the stability and inhibitory function of Tregs, which is highly correlated with poor clinical prognosis [190]. Therefore, treatment with anti-TIGIT mAbs can mediate the direct killing of tumor cells in patient tumor samples and preclinical mouse models and can also kill Tregs via Fc receptors (FCRs) [191].

In conclusion, after blocking ICR-ICL signaling with ICIs, γδT cells can gradually recover their functions and produce synergistic antitumor immune effects.

## Relationship between ICI resistance and γδT cells

The rate of response to ICIs is related to many factors [192, 193], and ICT can be a good option for cancer patients who are likely to have an active immune response. However, tumor cells may evolve other mechanisms of resistance after a period of treatment [78, 194–198]. The mechanisms of drug resistance may not be failure of targeted drugs but rather compensatory changes in γδT cells leading to acquired resistance to drugs [72, 199]. For example, in EGFR- and KRAS-mutant mice and human lung cancer specimens, therapeutic PD-1-blocking antibodies still bind to T cells as the disease progresses; this finding suggests that mAbs still play a role when drug resistance occurs, so attention should be given to pathways other than PD-1/PD-L1 in adaptive drug resistance [200].

During the course of treatment with mAbs, compensatory and alternative suppressor receptors on γδT cells are upregulated, indicating that γδT cells may also be involved in the development of ICI tolerance in cancer patients. Some data show that anti-PD-1 inhibition significantly increases the frequency of TIM-3+ Vδ2T cells, indicating compensatory upregulation of TIM-3; additionally, combined inhibition of TIM-3 and PD-1 can significantly increase the production of TNF-α and IFN-γ [97]. CTLA-4 and BTNL2 are also upregulated [126, 201], which proves that BTNL2 may also be part of a novel immune escape mechanism in cancer. In addition, in melanoma, anti-PD-1 monotherapy or anti-PD-1× anti-CTLA-4 antibodies combination therapy increases the expression of VISTA on lymphocytes and thus reshapes the tumor immune microenvironment, resulting in increased expression of PD-L1 in TAMs, increased Treg infiltration and decreased MHC expression on DCs; these results indicate that the tumor cells have acquired drug resistance, which is related to poor prognosis of the patient [202].

After the use of ICIs, the lack of the ICR signaling can promote γδT cells to secrete tumor-promoting cytokines and change toward tumor-promoting functional subgroups, suggesting that γδT cells are part of another ICI resistance mechanism. Studies have shown that a lack of the ICR signaling promotes the expression of IL-17 by γδT cells. For example, defects in the PD-1 gene promote the production of IL-17A and IL-22 by γδT cells [203]. Lack of BTLA activity further activates γδTh17 cells [204]. Lack of VISTA expression also increased the expression of IL-17A in γδTh17 cells [205]. IL-17A is a proinflammatory factor that can induce γδT cells to express various ICRs [98] and IL-17A-producing γδT cells may instigate resistance to ICT [206]. Furthermore, blocking TIM-3 significantly increases the expression of IL-21R and negatively regulates the antitumor function of γδT cells by promoting the development of CD73+ γδT cells [169]. In ICI therapy-resistant patients, there were higher proportions of TGD-c21 γδT cell subsets, and the ligand-receptor binding ability, IFN signal transduction and pathway activity of these cells were significantly reduced. Therefore, TGD-c21 cells might be exhausted γδT cells with impaired antitumor immune function [207]. In summary, the heterogeneity of γδT cell subsets can lead to a lack of response to immunotherapy. The interaction between ICs and cytokines and abnormalities in the IFN-γ signaling pathway can also lead to secondary drug resistance in the context of an inhibitory TME [27, 208].

Another acquired resistance mechanism is compensatory upregulation of other ICs. A preclinical study evaluated the efficacy of checkpoint blockade combined with anti-PD-1 and anti-BTLA therapy in the treatment of GBM [209], and the combined treatment effectively limited the progression of tumors. Therefore, multitarget blockade may better restore the antitumor function of γδT cells, but the clinical rationale remains to be further explored.

## The association between γδT cells and dysbiosis

Abnormal activation and increased numbers of γδT cells may induce immune-related adverse events (irAEs) or exacerbate preexisting autoimmune diseases of patients. As the immune system is activated, immune cells can become more active and secrete more proinflammatory cytokines, and this may lead to an increase in autoantibodies [210, 211], thus disrupting the balance between immunosuppression and immune activation. Studies have shown that irAEs caused by ICIs have a pathogenesis similar to that of autoimmune disorders, and hyperreactivity of Th17 cells and proinflammatory cytokines such as IL-17A, IL-21 and IL-22 have prominent roles in the pathogenesis of many autoimmune diseases [212]. Among these mechanisms, increased levels of IL-17 were found to be associated with the development of irAEs such as colitis after receipt of ICI therapy [213], suggesting that dysfunctional γδT17 cells may also be involved in the occurrence and development of irAEs. IrAEs often manifest as gastrointestinal toxicity, and adverse reactions such as celiac disease have been reported [214]. Researchers found that the number of γδT cells in patients with celiac disease was higher than that in normal people [215, 216], suggesting that abnormally activated γδT cells may contribute to gastrointestinal dysfunction.

Microbiota participate in immune regulation and can affect patient response to ICI therapy. γδT cells can interact with microbiota and influence antitumor immunity. Given their larger tumor size and shorter survival time, antibiotic-treated (Abt) mice are more susceptible to B16/F10 melanoma and Lewis lung carcinoma due to the loss of protection from nonspecific commensal microbiota. Abt mice also showed increased sensitivity to tumor development, accompanied by decreased expression of IL-17A, IL-6 and IL-23 by γδT17 cells and partially inhibited function of CD8+ αβT cells. Supplementation of γδT cells and IL-17 restored the antitumor immune response of Abt mice [217]. Paradoxically, after antibiotic treatment in mice that received antitumor immunotherapy, the amino acid metabolism pathway changed, and the increased production of 3-indole propionic acid enhanced the cytotoxicity of γδT cells by promoting the secretion of granzyme B, perforin and IFN-γ instead of IL-17 without affecting the proliferation of γδT cells [218].

Previous studies have shown that IL-17+ γδT cells participate in dysbiosis, and IL-17+ γδT cells may be the effector T cells that regulate intestinal dysbiosis. *Escherichia coli* can promote the phagocytosis and killing capacity of γδT cells in a TCR-dependent manner [219]. After the onset of dysbiosis induced by ischemic brain injury, Th17 cells are not significantly affected, while the frequency of γδT17 cells in the intestinal tract is altered, and these cells are involved in injury repair and neuroprotection after homeostatic dysregulation [220]. Additionally, the proportion of γδT cells in the liver is 5–10 times higher than that in other tissues and organs, and some studies have suggested a tissue-specific interaction between the microbiota and immune cells in the liver. Hepatocyte-expressed CD1d can present lipid antigens from microbiota, stimulate TCRγδ and maintain homeostasis of liver-resident γδT17 cells. Moreover, in nonalcoholic fatty liver disease, an overall high microbiota load can induce an increase in hepatic γδT17 numbers, which may be accompanied by a bystander effect. For example, inflammation induced by a high-fat diet (HFD) can stimulate γδT17 cells, and together, these factors exacerbate disease progression [221].

Microbiota and their metabolites affect the proliferation, cytokine secretion and cytotoxicity of γδT cells, and the interactions among γδT cells, microbiota and other immune cells are closely related to the maintenance of homeostasis and have significance for immune surveillance.

## Future perspective

The binding of ICLs and ICRs is a means by which tumor cells escape immunity, and it is also the chief cause of the dysfunction of γδT cells. However, the relationship between γδT-cell-targeting ICIs and the TME is rarely discussed. ICT based on traditional cytotoxic T lymphocytes commonly has a low response rate due to MHC restriction. Therefore, it is important to exploit the antitumor potential of γδT cells. In regard to the relationship between γδT-cell-targeting ICIs and the TME, there are still many topics worthy of further study.

The heterogeneity of γδT-cell subsets needs to be further clarified. There are many subpopulations of γδT cells, but there is some overlap between subgroups characterized based on structural classifications and functional classifications. Moreover, γδT cells have high plasticity, and their specific functional state is easily controlled by the TME. Therefore, it is necessary to reveal the relationships between surface markers and functional changes in γδT cells with the help of techniques such as single-cell and spatial omics analyses [222]. For example, the upregulation of ICs does not mean the absolute exhaustion of γδT cells [141, 143]. As such, it remains to be determined how the specific epigenetic state of γδT cells changes as dysfunction develops. Furthermore, when chromatin remodeling occurs, is it reversible? How do γδT cells exhibit opposite effects under the influence of different TMEs, and can these effects be exploited? Are the changes in the expression patterns of γδT-cell ICs similar to those of αβT cells? If future research can identify a method for selective targeting of certain subsets, γδT cells will be able to play a more efficient antitumor role.

Tumor models used for the study of γδT cells urgently need to be improved for several reasons. First, the groups of γδT cells are different between humans and mice [37]. Therefore, the subgroup results obtained in mice cannot be perfectly applied to humans. Second, BTNs similar to those in humans have not been found in mice. Therefore, rodent models cannot completely represent the actual environment in humans [223]. Furthermore, the TME is different in different tumor models and is also different from that in humans.

The therapeutic prospects of targeting checkpoints on the surface of γδT cells are broad. However, resistance to ICT based on γδT can occur, which necessitates researches on the mechanism underlying resistance to γδT-cell therapy and multitarget combination therapy. Furthermore, the role of γδT cells in irAEs needs to be further clarified, which will pave the way for the inclusion of γδT cells in the arsenal of immunotherapy options.

First, MHC molecule independence and glycolipid antigen recognition activity are great advantages for γδT cells. In ICI-resistant tumor cells, a similarity between cells with ICI resistance and a hypermetabolic phenotype was observed. This phenotype included synergistic upregulation of glycolysis and mitochondrial oxidative phosphorylation, which assists tumor cells in maintaining vigorous growth under hypoxia and resisting ICT [195, 224]. The associations between checkpoint molecules related to the internal metabolism of tumor cells and ICs is also an interesting topic [225].

Second, ICs can be either costimulatory or coinhibitory molecules. Agents targeting inhibitory molecules combined with αβT cell agonists have been shown to have synergistic antitumor effects [226–230]. As dysfunctional γδT cells can also express multiple ICs, it may be possible to restore the antitumor potential of γδT cells. In addition, given that the mechanism of action of CD39/CD73 is relatively independent of traditional ICs and that CD39/CD73 and its related ADO pathway have been proven to be related to the survival and prognosis of patients [231], the rationality of ICI combined with other targets, such as agents targeting CD39/CD73, deserves further discussion.

In addition to considering the combination of multitarget therapy with ICT, whether combination immunotherapy based on γδT cells can have a synergistic effect is also worth exploring. Vγ9Vδ2T cells can release a large number of cytokines and chemokines to induce the activation of other bystander immune cells after being activated by amino bisphosphonic acids such as ZA. With the help of MCP-2, γδT cells can activate the effector function of granulocytes [232]. Some studies have shown that the maturation of immature DCs may mainly result from a bystander process because immature DCs rely on the activation of Vγ9Vδ2T cells by TCR signal transduction and are able to promote Vγ9Vδ2T cells to secrete cytokines required for the maturation process [233]. Bystander activation of T cells is a type of antigen-independent activation that can occur through different receptors and costimulatory signals and has been observed in cancers [234]. Microorganism-related pattern recognition receptors, such as Toll-like receptors (TLRs), and cytokines such as IL-12, IL-15, and IL-18 are associated with activating bystander T-cell responses [235]. Thus, both the innate immune properties of γδT cells and their excellent cytokine and chemokine secretion abilities may aid in the recruitment of other immune cells [236] or interactions with other bystander cells in the TME and may be involved in bystander activation. Bystander T cells rarely show an exhausted status and have excellent innate killing ability [237]; therefore, reversing the immunosuppressive state of γδT cells may allow γδT cells to promote ICI responsiveness by activating bystander cells. ICT based on γδT cells combined with strategies that target bystander T cells (such as intratumoral injection of viral peptides) may have additional beneficial effects on antitumor immunity, but this hypothesis needs to be further experimentally verified and mechanistically explored.

In clinical applications, adoptive cell infusion based on γδT cells combined with ICIs or CAR-γδT cells may be promising future research directions. The feasibility and antitumor ability of CAR-γδT cells have been preliminarily proven [238, 239], and it has been shown that CAR-γδT cells can successfully migrate into the TME and cross-present tumor-associated antigens, providing more stable and durable antitumor immunity than conventional CAR-T cells in the solid tumor environment [240–242]. Compared with CAR-αβT cells, CAR-γδT cells have no alloreactivity and lower incidences of off-target toxicities and cytokine release syndrome [243, 244]. Arming CAR-T cells with bacterial-derived virulence factors with strong immunomodulatory properties can mediate bystander immunity through epitope spreading and can expand the therapeutic spectrum [245]. Antigen recognition by γδT cells is closely related to microbial metabolites, indicating that CAR-γδT cells may benefit from microbial engineering. CAR-γδT cells have just taken the first step toward clinical application [246, 247], and more clinical trials are required to determine the potential of CAR-γδT cells. In clinical trials, excellent antitumor activity and good patient tolerance were demonstrated for adoptive γδT-cell-based therapies after γδT cells were expanded in the presence of appropriate doses of ZA or BrHPP or infused with IL-2/IL-21 [248]. One study showed that Vδ2T cells were able to maintain a low PD-1 expression state in vivo after administration of immunotherapy [249]. However, not all patients respond to adoptive Vγ9Vδ2T-cell therapies. Considering that γδT cells present in the suppressive TME can display exhaustion or anergy, combination with ICIs might improve the cytotoxicity of γδT-cell immunotherapy and lead to better antitumor effects [250].

First describing the interaction between the TME and γδT cells, this paper has summarized the potential of γδT cells to participate in ICT by describing the functional regulation of ICs on γδT cells. Agents targeting ICs can significantly regulate the proliferation, activation and cytotoxicity of γδT cells, which provides a strategy to reverse the immunosuppressive state of γδT cells and supports the use of ICT based on γδT cells in clinical applications in the future.

## Supplementary Information

**Additional file 1: Fig. S1.** Heatmap of γδT cells in TCGA dataset. The heatmap is showing characteristic distributions of pan-γδT cells and other γδT-cell subgroups (column labels) in different types of cancers (row labels).

**Additional file 2: Fig. S2.** Heatmap of γδT cells in GTEx dataset. The heatmap is showing characteristic distributions of pan-γδT cells and other γδT-cell subgroups (column labels) in different types of tissues (row labels).

**Additional file 3: Fig. S3.** Anatomical heatmaps of γδT cells in TCGA dataset. Anatomical heatmaps exhibit enrichment scores of γδT cells and other subtypes, including pan-γδT cell, Vγ9Vδ2T cells, cytotoxic γδT cells, IFN-γ-producing γδT cells, γδNKT cells, type2-like γδT cells, γδT17 cells and γδTregs across given anatomic locations.

**Additional file 4: Fig. S4.** Anatomical heatmaps of γδT cells in GTEx dataset. Anatomical heatmaps exhibit enrichment scores of γδT cells and other subtypes, including pan-γδT cell, Vγ9Vδ2T cells, cytotoxic γδT cells, IFN-γ-producing γδT cells, γδNKT cells, type2-like γδT cells, γδT17 cells and γδTregs across given anatomic locations.

**Additional file 5: Supplememtary Table 1.**

**Additional file 6.**

## Data Availability

Not applicable.

## Abbreviations

Abt: Antibiotic-treated
ADCC: Antibody-dependent cell-mediated cytotoxicity
ADO: Adenosine
AML: Acute myelogenous leukemia
AMP: Adenosine monophosphate
A2AR: A2A receptor
BrHPP: Bromohydrin pyrophosphate
BTLA: B- and T-lymphocyte attenuator
BTN2A: Butyrophilin 2A
BTN3A: Butyrophilin 3A
CAR: Chimeric antigen receptor
CCL2: C-C motif chemokine 2
CTLA-4: Cytotoxic T-lymphocyte-associated protein 4
DNT: Double-negative T cell
eATP: Extracellular adenosine triphosphate
FCR: Fc receptor
GBM: Glioblastoma
GC: Germinal center
G-CSF: Granulocyte colony-stimulating factor
GM-CSF: Granulocyte-macrophage colony-stimulating factor
HIF: Hypoxia-inducible factor
HMBPP: (e)-4-hydroxy-3-methyl-but-2-enylpyrophosphate
HMGB-1: High mobility group box protein 1
IC: Immune checkpoint
ICB: Immune checkpoint blockade
ICI: Immune checkpoint inhibitor
ICL: Immune checkpoint ligand
ICR: Immune checkpoint receptor
IFN-γ: Interferon-gamma
IL: Interleukin
ILT2: Ig-like transcript 2
IPP: Isopentenyl pyrophosphate
ITIM: Immune receptor tyrosine-based inhibition motif
irAEs: Immune-related adverse events
ITSM: Immune receptor tyrosine-based switch motif
LAG-3: Lymphocyte-activation gene 3
MCP-1: Monocyte chemoattractant protein 1
MCP-2: Monocyte chemoattractant protein 2
MHC: Major histocompatibility complex
MM: Multiple myeloma
MDSC: Myeloid-derived suppressor cell
OS: Overall survival
TAM: Tumor-associated macrophage
TCR: T-cell receptor
TGF: Transforming growth factor-beta
TIM-3: T-cell immunoglobulin domain and mucin domain-3
TIL: Tumor-infiltrating lymphocyte
TLR: Toll-like receptor
TME: Tumor microenvironment
TMG: Tumor marker ganglioside
TNF-α: Tumor necrosis factor-alpha
PAg: Phosphoantigen
PB: Peripheral blood
PDAC: Pancreatic ductal adenocarcinoma
pDC: Plasmacytoid dendritic cell
pMHC: Peptide-MHC-I complexes
ROS: Reactive oxygen species
SHM: Somatic hypermutation
SHP: Scr homology region 2 domain-containing phosphatase
Siglec: Sialic acid binding immunoglobulin-like lectin
STAT: Signal transducer and activator of transcription
ZA: Zoledronic acid
