# Supplementary figures and images for: Gamma delta T-cell-based immune checkpoint therapy: attractive candidate for antitumor treatment
Source: Mol Cancer. 2023 Feb 15;22:31. doi: 10.1186/s12943-023-01722-0 (PMC9930367; doi:10.1186/s12943-023-01722-0)

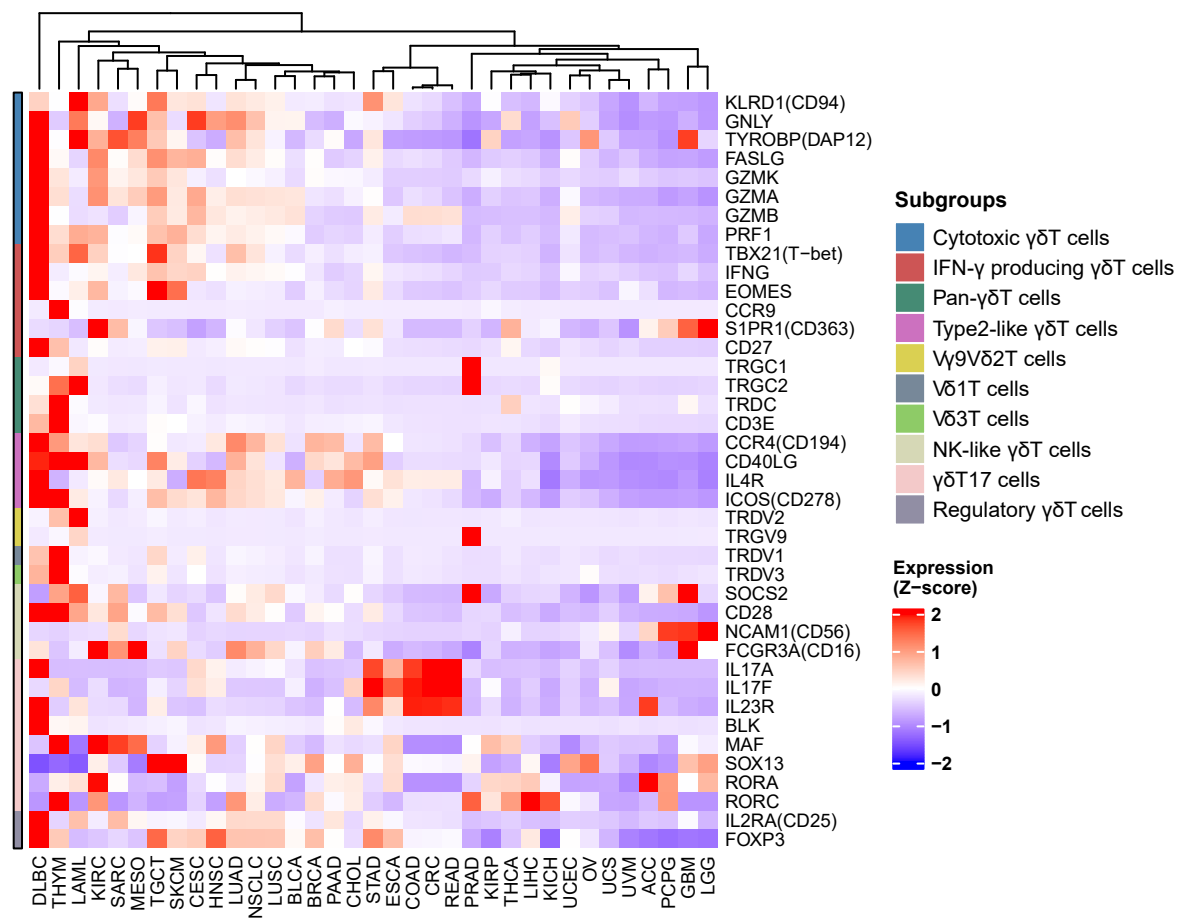

Supplement: Supplementary file 1 — Additional file 1: Fig. S1. Heatmap of γδT cells in TCGA dataset. The heatmap is showing characteristic distributions of pan-γδT cells and other γδT-cell subgroups (column labels) in different types of cancers (row labels). [file 12943_2023_1722_MOESM1_ESM.pdf]

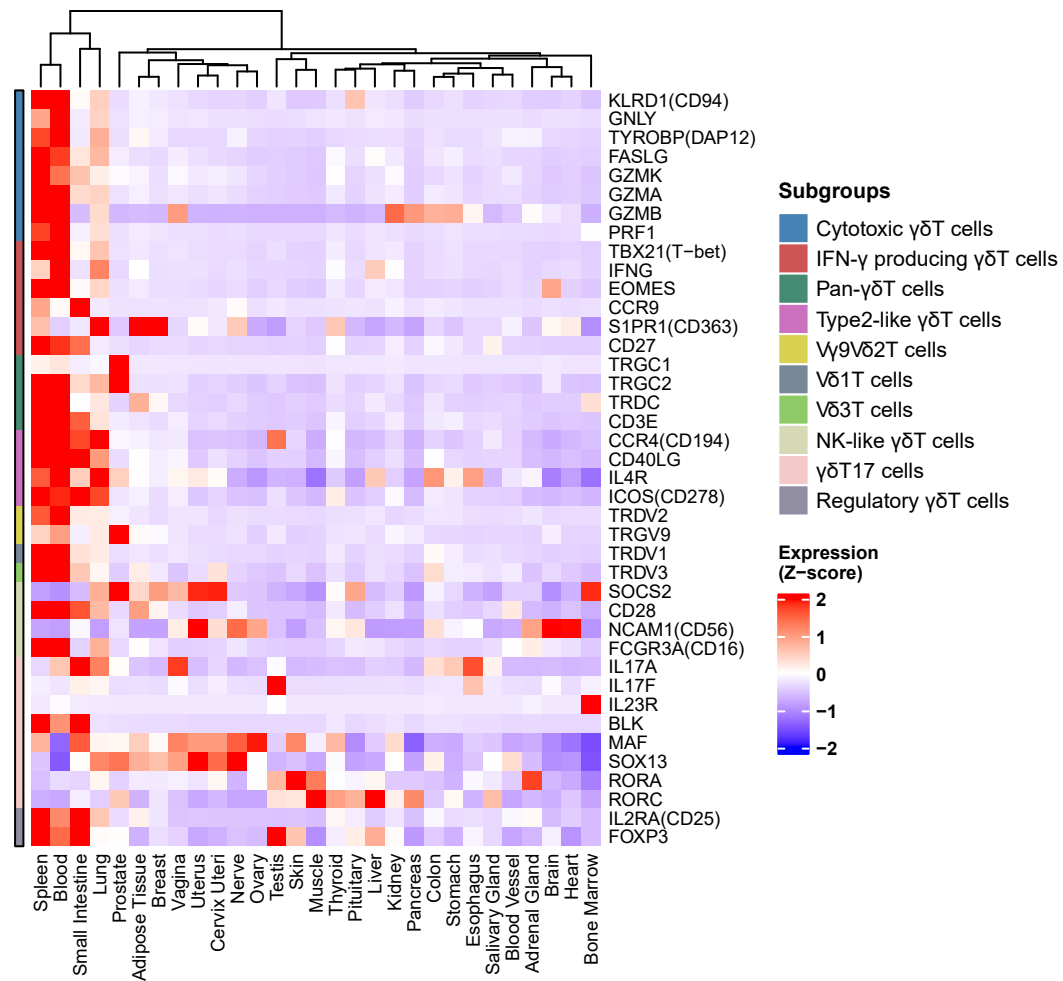

Supplement: Supplementary file 2 — Additional file 2: Fig. S2. Heatmap of γδT cells in GTEx dataset. The heatmap is showing characteristic distributions of pan-γδT cells and other γδT-cell subgroups (column labels) in different types of tissues (row labels). [file 12943_2023_1722_MOESM2_ESM.pdf]

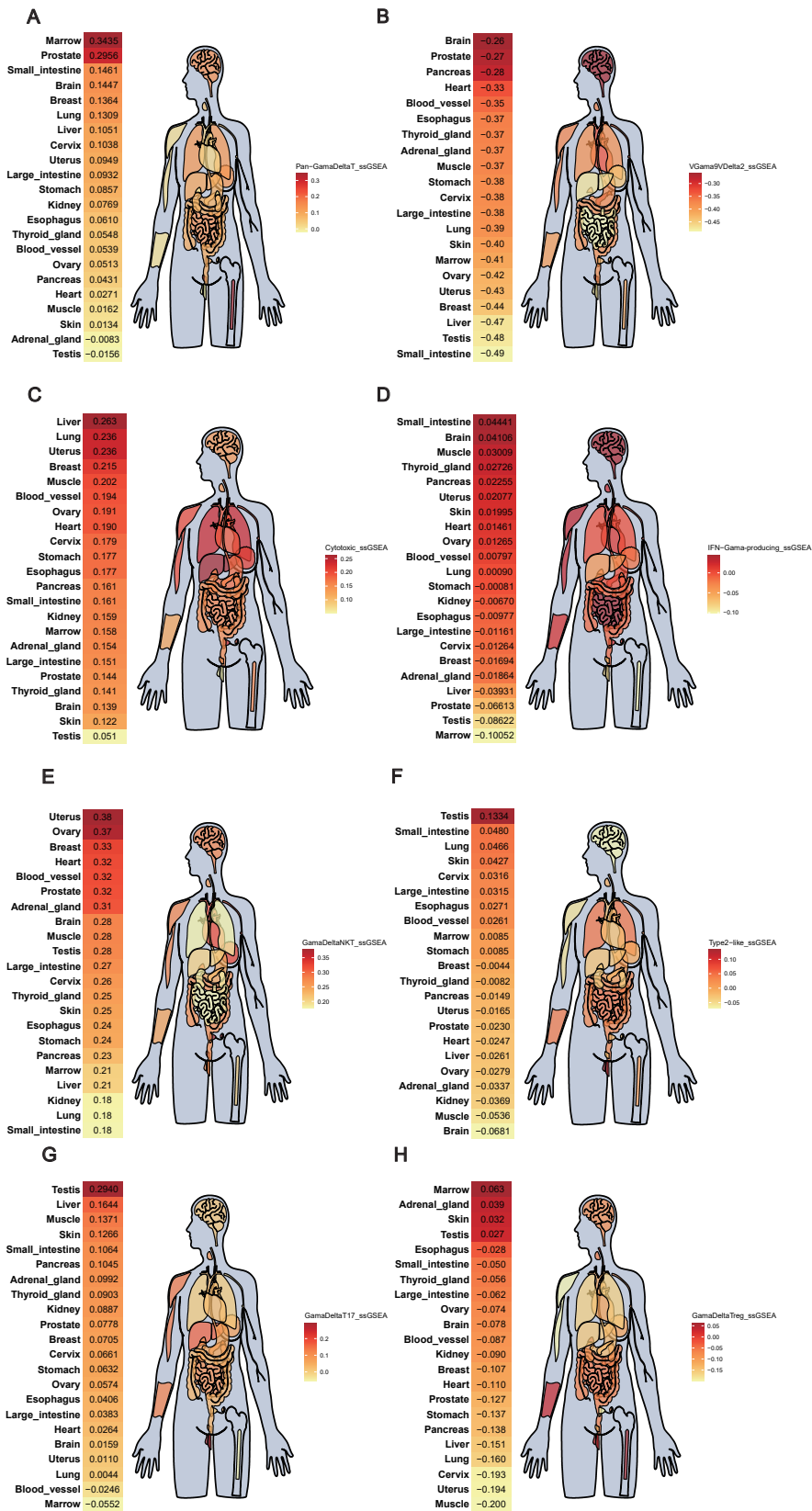

Supplement: Supplementary file 4 — Additional file 4: Fig. S4. Anatomical heatmaps of γδT cells in GTEx dataset. Anatomical heatmaps exhibit enrichment scores of γδT cells and other subtypes, including pan-γδT cell, Vγ9Vδ2T cells, cytotoxic γδT cells, IFN-γ-producing γδT cells, γδNKT cells, type2-like γδT cells, γδT17 cells and γδTregs across given anatomic locations. [file 12943_2023_1722_MOESM4_ESM.pdf]
