# Supplementary material for: Gamma delta T-cell-based immune checkpoint therapy: attractive candidate for antitumor treatment
Source: Mol Cancer. 2023 Feb 15;22:31. doi: 10.1186/s12943-023-01722-0 (PMC9930367; doi:10.1186/s12943-023-01722-0)
Supplement: Supplementary file 3 — Additional file 3: Fig. S3. Anatomical heatmaps of γδT cells in TCGA dataset. Anatomical heatmaps exhibit enrichment scores of γδT cells and other subtypes, including pan-γδT cell, Vγ9Vδ2T cells, cytotoxic γδT cells, IFN-γ-producing γδT cells, γδNKT cells, type2-like γδT cells, γδT17 cells and γδTregs across given anatomic locations. [file 12943_2023_1722_MOESM3_ESM.pdf]

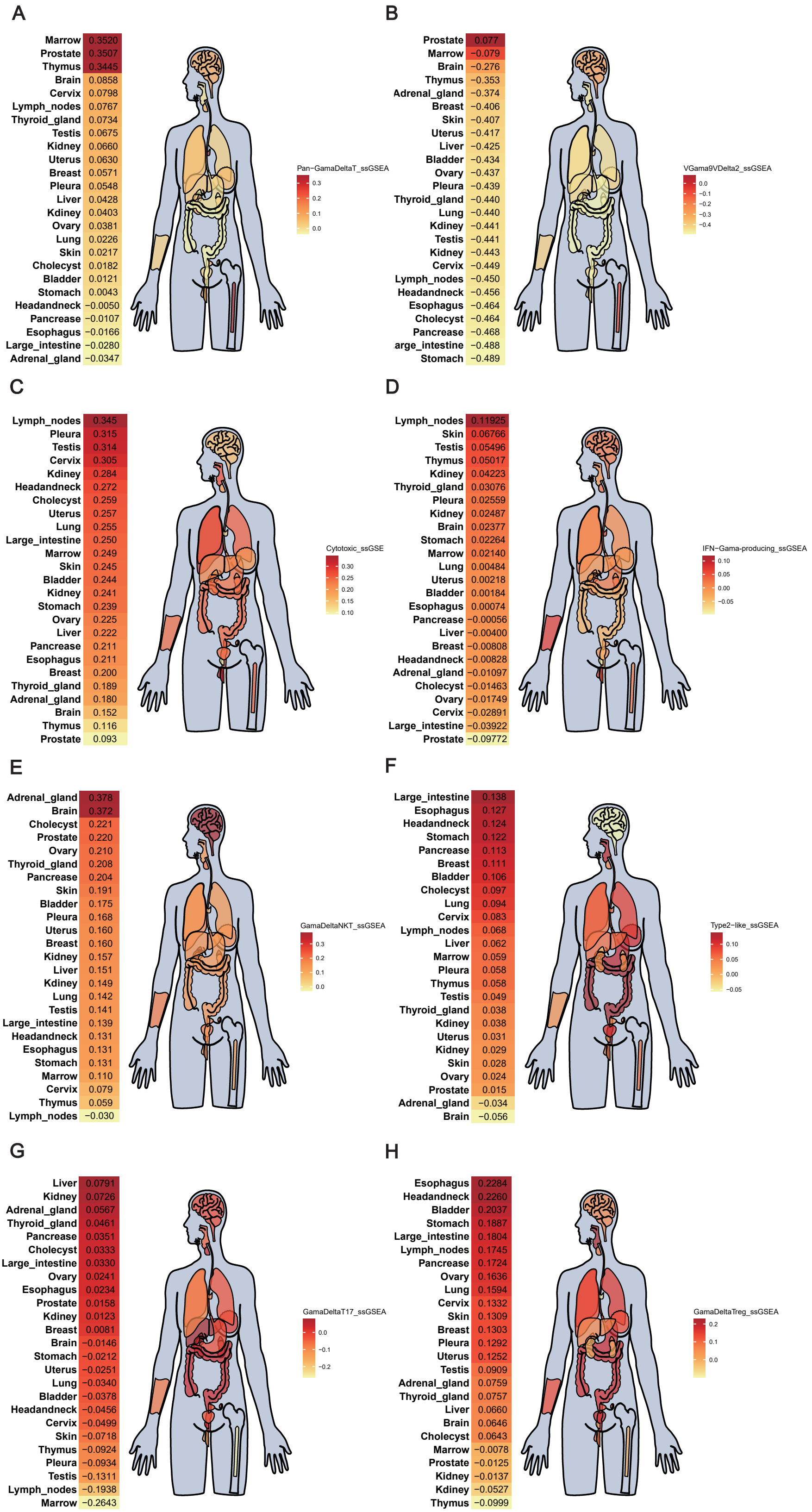
A
Marrow 0.3520
Prostate 0.3507
Thymus 0.3445
Brain 0.0858
Cervix 0.0798
Lymph_nodes 0.0767
Thyroid_gland 0.0734
Testis 0.0675
Kidney 0.0660
Uterus 0.0630
Breast 0.0571
Pleura 0.0548
Liver 0.0428
Kdiney 0.0403
Ovary 0.0381
Lung 0.0226
Skin 0.0217
Cholecyst 0.0182
Bladder 0.0121
Stomach 0.0043
Headandneck −0.0050
Pancrease −0.0107
Esophagus −0.0166
Large_intestine −0.0280
Adrenal_gland −0.0347
Pan−GamaDeltaT_ssGSEA
0.3
0.2
0.1
0.0
B
Prostate 0.077
Marrow −0.079
Brain −0.276
Thymus −0.353
Adrenal_gland −0.374
Breast −0.406
Skin −0.407
Uterus −0.417
Liver −0.425
Bladder −0.434
Ovary −0.437
Pleura −0.439
Thyroid_gland −0.440
Lung −0.440
Kdiney −0.441
Testis −0.441
Kidney −0.443
Cervix −0.449
Lymph_nodes −0.450
Headandneck −0.456
Esophagus −0.464
Cholecyst −0.464
Pancrease −0.468
arge_intestine −0.488
Stomach −0.489
VGama9VDelta2_ssGSEA
0.0
−0.1
−0.2
−0.3
−0.4
C
Lymph_nodes 0.345
Pleura 0.315
Testis 0.314
Cervix 0.305
Kdiney 0.284
Headandneck 0.272
Cholecyst 0.259
Uterus 0.257
Lung 0.255
Large_intestine 0.250
Marrow 0.249
Skin 0.245
Bladder 0.244
Kidney 0.241
Stomach 0.239
Ovary 0.225
Liver 0.222
Pancrease 0.211
Esophagus 0.211
Breast 0.200
Thyroid_gland 0.189
Adrenal_gland 0.180
Brain 0.152
Thymus 0.116
Prostate 0.093
Cytotoxic_ssGSE
0.30
0.25
0.20
0.15
0.10
D
Lymph_nodes 0.11925
Skin 0.06766
Testis 0.05496
Thymus 0.05017
Kdiney 0.04223
Thyroid_gland 0.03076
Pleura 0.02559
Kidney 0.02487
Brain 0.02377
Stomach 0.02264
Marrow 0.02140
Lung 0.00484
Uterus 0.00218
Bladder 0.00184
Esophagus 0.00074
Pancrease −0.00056
Liver −0.00400
Breast −0.00808
Headandneck −0.00828
Adrenal_gland −0.01097
Cholecyst −0.01463
Ovary −0.01749
Cervix −0.02891
Large_intestine −0.03922
Prostate −0.09772
IFN−Gama−producing_ssGSEA
0.10
0.05
0.00
−0.05
E
Adrenal_gland 0.378
Brain 0.372
Cholecyst 0.221
Prostate 0.220
Ovary 0.210
Thyroid_gland 0.208
Pancrease 0.204
Skin 0.191
Bladder 0.175
Pleura 0.168
Uterus 0.160
Breast 0.160
Kidney 0.157
Liver 0.151
Kdiney 0.149
Lung 0.142
Testis 0.141
Large_intestine 0.139
Headandneck 0.131
Esophagus 0.131
Stomach 0.131
Marrow 0.110
Cervix 0.079
Thymus 0.059
Lymph_nodes −0.030
GamaDeltaNKT_ssGSEA
0.3
0.2
0.1
0.0
F
Large_intestine 0.138
Esophagus 0.127
Headandneck 0.124
Stomach 0.122
Pancrease 0.113
Breast 0.111
Bladder 0.106
Cholecyst 0.097
Lung 0.094
Cervix 0.083
Lymph_nodes 0.068
Liver 0.062
Marrow 0.059
Pleura 0.058
Thymus 0.058
Testis 0.049
Thyroid_gland 0.038
Kdiney 0.038
Uterus 0.031
Kidney 0.029
Skin 0.028
Ovary 0.024
Prostate 0.015
Adrenal_gland −0.034
Brain −0.056
Type2−like_ssGSEA
0.10
0.05
0.00
−0.05
G
Liver 0.0791
Kidney 0.0726
Adrenal_gland 0.0567
Thyroid_gland 0.0461
Pancrease 0.0351
Cholecyst 0.0333
Large_intestine 0.0330
Ovary 0.0241
Esophagus 0.0234
Prostate 0.0158
Kdiney 0.0123
Breast 0.0081
Brain −0.0146
Stomach −0.0212
Uterus −0.0251
Lung −0.0340
Bladder −0.0378
Headandneck −0.0456
Cervix −0.0499
Skin −0.0718
Thymus −0.0924
Pleura −0.0934
Testis −0.1311
Lymph_nodes −0.1938
Marrow −0.2643
GamaDeltaT17_ssGSEA
0.0
−0.1
−0.2
H
Esophagus 0.2284
Headandneck 0.2260
Bladder 0.2037
Stomach 0.1887
Large_intestine 0.1804
Lymph_nodes 0.1745
Pancrease 0.1724
Ovary 0.1636
Lung 0.1594
Cervix 0.1332
Skin 0.1309
Breast 0.1303
Pleura 0.1292
Uterus 0.1252
Testis 0.0909
Adrenal_gland 0.0759
Thyroid_gland 0.0757
Liver 0.0660
Brain 0.0646
Cholecyst 0.0643
Marrow −0.0078
Prostate −0.0125
Kidney −0.0137
Kdiney −0.0527
Thymus −0.0999
GamaDeltaTreg_ssGSEA
0.2
0.1
0.0
