# Supplementary material for: Gamma delta T-cell-based immune checkpoint therapy: attractive candidate for antitumor treatment
Source: Mol Cancer. 2023 Feb 15;22:31. doi: 10.1186/s12943-023-01722-0 (PMC9930367; doi:10.1186/s12943-023-01722-0)
Supplement: Supplementary file 6 — Additional file 6. [file 12943_2023_1722_MOESM6_ESM.pdf]

Supplementary information

To better elucidate the tissue specificity of γδT cells, we added additional analyses and presented the results in the form of heatmaps (Supplementary Figure 1-2). We downloaded RNA-seq data (Fragments Per Kilobase of exon model per Million mapped fragments, FPKM) for 33 cancer types from the TCGA database (https://portal.gdc.cancer.gov/) [1] and merged the data of lung adenocarcinoma (LUAD) and lung squamous cell carcinoma (LUSC) into non-small cell lung cancer (NSCLC), colon adenocarcinoma (COAD) and rectum adenocarcinoma (READ) into colorectal cancer (CRC). In addition, we downloaded Genotype-Tissue Expression Project (GTEx) RNA-seq data (FPKM) from the XENA database (https://xenabrowser.net/datapages/) [2]. We collated the marker genes of different γδT cells from the published literature (Supplementary Table 1) [3–13]. We used single sample gene set enrichment analysis (ssGSEA) for each cancer type or each type of tissue regarding the signatures of different γδT cells [14]. In addition, we showed the scores of pan-γδT cells and other γδT-cell subgroups of different organs in GTEx and TCGA databases respectively in the anatomical heatmaps (Supplementary Figure 3-4), which were produced through MOAHIT, a web tool for visualizing human anatomical data [15]. The ComplexHeatmap R package was used to visualize the heatmaps [16].

References

1. Tomczak K, Czerwińska P, Wiznerowicz M. The Cancer Genome Atlas (TCGA): an immeasurable source of knowledge. Contemp Oncol (Pozn). 2015;19:A68-77.

2. Goldman M, Craft B, Hastie M, Repečka K, McDade F, Kamath A, et al. The UCSC Xena platform for public and private cancer genomics data visualization and interpretation [Internet]. bioRxiv; 2019 [cited 2022 Dec 30]. p. 326470. Available from: https://www.biorxiv.org/content/10.1101/326470v6

3. Pizzolato G, Kaminski H, Tosolini M, Franchini D-M, Pont F, Martins F, et al. Single-cell RNA sequencing unveils the shared and the distinct cytotoxic hallmarks of human TCRVδ1 and TCRVδ2 γδ T lymphocytes. Proc Natl Acad Sci U S A. 2019;116:11906–15.

4. Loh EY, Cwirla S, Serafini AT, Phillips JH, Lanier LL. Human T-cell-receptor delta chain: genomic organization, diversity, and expression in populations of cells. Proc Natl Acad Sci U S A. 1988;85:9714–8.

5. Lefranc MP, Rabbitts TH. The human T-cell receptor gamma (TRG) genes. Trends Biochem Sci. 1989;14:214–8.

6. Allison TJ, Winter CC, Fournié JJ, Bonneville M, Garboczi DN. Structure of a human gammadelta T-cell antigen receptor. Nature. 2001;411:820–4.

7. Kazen AR, Adams EJ. Evolution of the V, D, and J gene segments used in the primate gammadelta T-cell receptor reveals a dichotomy of conservation and diversity. Proc Natl Acad Sci U S A. 2011;108:E332-340.

8. Boufea K, González-Huici V, Lindberg M, Symeonides S, Oikonomidou O, Batada NN. Single-cell RNA sequencing of human breast tumour-infiltrating immune cells reveals a γδ T-cell subtype associated with good clinical outcome. Life Sci Alliance. 2021;4:e202000680.

9. Sanchez Sanchez G, Papadopoulou M, Azouz A, Tafesse Y, Mishra A, Chan JKY, et al. Identification of distinct functional thymic programming of fetal and pediatric human γδ thymocytes via single-cell analysis. Nat Commun. 2022;13:5842.

10. Hu Y, Fang K, Wang Y, Lu N, Sun H, Zhang C. Single-cell analysis reveals the origins and intrahepatic development of liver-resident IFN-γ-producing γδ T cells. Cell Mol Immunol. 2021;18:954–68.

11. Sagar null, Pokrovskii M, Herman JS, Naik S, Sock E, Zeis P, et al. Deciphering the regulatory landscape of fetal and adult γδ T-cell development at single-cell resolution. EMBO J. 2020;39:e104159.

12. Tan L, Sandrock I, Odak I, Aizenbud Y, Wilharm A, Barros-Martins J, et al. Single-Cell Transcriptomics Identifies the Adaptation of Scart1+ Vγ6+ T Cells to Skin Residency as Activated Effector Cells. Cell Rep. 2019;27:3657-3671.e4.

13. Casetti R, Agrati C, Wallace M, Sacchi A, Martini F, Martino A, et al. Cutting edge: TGF-beta1 and IL-15 Induce FOXP3+ gammadelta regulatory T cells in the presence of antigen stimulation. J Immunol. 2009;183:3574–7.

14. Hänzelmann S, Castelo R, Guinney J. GSVA: gene set variation analysis for microarray and RNA-seq data. BMC Bioinformatics. 2013;14:7.

15. Zhou C, Lin A, Zhang J, Luo P. MOAHIT: a web tool for visualizing tumor multi-omics data with human anatomy heatmaps [Internet]. bioRxiv; 2022 [cited 2022 Dec 30]. p. 2022.09.07.506938. Available from: https://www.biorxiv.org/content/10.1101/2022.09.07.506938v2

16. Gu Z, Eils R, Schlesner M. Complex heatmaps reveal patterns and correlations in multidimensional genomic data. Bioinformatics. 2016;32:2847–9.
